# Model-based exploration of the impact of glucose metabolism on the estrous cycle dynamics in dairy cows

**DOI:** 10.1186/s13062-019-0256-7

**Published:** 2020-01-15

**Authors:** Mohamed Omari, Alexander Lange, Julia Plöntzke, Susanna Röblitz

**Affiliations:** 1 Computational Systems Biology Group, Zuse Institute Berlin, Takustr. 7, Berlin, Germany; 2 Department of Applied Biosciences and Process Engineering, Anhalt University of Applied Sciences, Bernburger Str. 55, Köthen, 06366 Germany; 3 Computational Biology Unit, University of Bergen, Department of Informatics, Thormøhlensgate 55, Bergen, 5008 Norway

**Keywords:** Systems biology, Mathematical modelling, Ordinary differential equations, Metabolism, Nutrition, Bovine, Fertility, Reproduction, Lactation, Hormones, Follicles

## Abstract

**Background:**

Nutrition plays a crucial role in regulating reproductive hormones and follicular development in cattle. This is visible particularly during the time of negative energy balance at the onset of milk production after calving. Here, elongated periods of anovulation have been observed, resulting from alterations in luteinizing hormone concentrations, likely caused by lower glucose and insulin concentrations in the blood. The mechanisms that result in a reduced fertility are not completely understood, although a close relationship to the glucose-insulin metabolism is widely supported.

**Results:**

Following this idea, we developed a mathematical model of the hormonal network combining reproductive hormones and hormones that are coupled to the glucose compartments within the body of the cow. The model is built on ordinary differential equations and relies on previously introduced models on the bovine estrous cycle and the glucose-insulin dynamics. Necessary modifications and coupling mechanisms are thoroughly discussed. Depending on the composition and the amount of feed, in particular the glucose content in the dry matter, the model quantifies reproductive hormones and follicular development over time. Simulation results for different nutritional regimes in lactating and non-lactating dairy cows are examined and compared with experimental studies. The simulations describe realistically the effects of nutritional glucose supply on the ovulatory cycle of dairy cattle.

**Conclusions:**

The mathematical model enables the user to explore the relationship between nutrition and reproduction by running simulations and performing parameter studies. Regarding its applicability, this work is an early attempt towards developing in silico feeding strategies and may eventually help to refine and reduce animal experiments.

**Reviewers:**

This article was reviewed by John McNamara and Tin Pang (nominated by Martin Lercher).

## Background

A few weeks after calving, modern high-yielding dairy cattle in intensive production systems give around 40 liters of milk per day. This is a high amount that comes at a cost. High-producing cows are highly susceptible to diseases, show metabolic disorders and fertility problems [1]. Early culling and smaller lifetime milk production are the consequences [2]. Countermeasures have already been taken, and the trend of breeding cows with ever increasing peak milk yield – prevalent for decades – may have come to an end. Optimizing lifetime milk production has proven to be more beneficial for both economic as well as environmental reasons [3].

The most critical time period for a cow’s health and her future performance is the periparturient period and the period of early lactation [4, 5]. During that time, the cow mobilizes body reserves because of her inability to meet energy demands solely from the feed energy consumed. This state is referred to as negative energy balance (NEB) [6].

For ruminants, the energy content in feed cannot be increased without limits due to the fermentative character of the digestive system [7]. High energetic feed with little fiber leads to an imbalance of microbes, rumen acidosis, and may even cause severe illness and death. Nevertheless, targeted feeding strategies are able to extenuate the NEB and to ensure animal health and welfare [5, 8, 9].

A number of experimental and clinical studies were performed to examine the relationship between the metabolic status and the fertility of cows, both, in qualitative and in quantitative manners, e.g., [10, 11]. Reduced nutrition intake was observed to delay the onset of puberty in beef heifers [12–14], to change the growth pattern of the dominant follicles (maximal diameter, persistence, number of follicular waves) [15], and to increase the period to conception postpartum [16–18]. Studies in the postpartum period of dairy cows showed that the NEB is strongly correlated with low concentrations of glucose, insulin and IGF-1 in the blood [19–21]. Changes in the secretion of gonadotropins, caused by low glucose levels, lead to low FSH and LH concentrations [10, 22], whereby missing LH peaks cause anovulation [4]. Non-regular estrous cycles are often associated with low average concentrations of insulin in the blood [23]. On the other hand, it was reported that good feed management, e.g., nutritional manipulation that causes increased insulin, reduces the incidence of non-regular estrous cycles [24].

This paper focuses on glucose, as part of the feed and as one of the main energy sources of the body. The aim is to develop a mathematical model that represents metabolic processes as well as reproductive regulation, thus allowing to analyze the impact of glucose originating from the feed on the reproductive hormones and the follicular development.

Previous modeling efforts mainly focused on either the bovine estrous cycle [25–29] or the nutritional strategies [30–32], yet there are a few approaches that combine the two topics. The most recent model, named “Jenny”, was developed by McNamara and Shields [33]. It connects the reproductive cycle (given by differential equations from [25, 26]) with nutrition (implemented by a rather sophisticated model called Molly [31]) via the ATP to ADP reduction reaction. Martin et al. [34] introduced an empirical model that includes nutritional effects on the reproduction. Pring et al. [27] modeled different nutritional scenarios by varying parameters in an estrous cycle model. A more conceptual model was suggested by Scaramuzzi et al. [35], where the coupling between nutrition and reproduction is realized by IGF-1, the glucose-insulin system, and leptin.

None of these models, except [33], captures the dynamics between nutrition, hormonal regulation, and milk yield, mechanisms that are of particular interest in cows. The model of McNamara and Shields [33] contains these elements, as it is based not only on Molly but on the BovCycle model [25, 26]. The effort by McNamara and Shields and the effort here are closely related and complementary. However, McNamara and Shields [33] do not include the full reproductive process. The model introduced here aims at understanding the involved interactions and time evolution on a more detailed level. It includes compartments for the nutrient intake, the glucose-insulin system [36], the milk production, and the reproductive hormones [26]. Based on that model, it is analyzed how changes in dietary intake, which usually happen on the time-scale of days, affect the behavior of the estrous cycle on the scale of weeks and months.

The paper is organized as follows. The glucose-insulin model and its coupling to the estrous cycle model are presented in the “Methods” section. The “Results and discussion” section deals with the simulation for non-lactating and lactating cows and compares the outcome with data from literature. Finally, the results are summarized again and limitations of the model are presented in the Conclusion. The model was implemented in MATLAB (release 2014b). The code is available in Additional file 1.

## Methods

The model that is developed in this section and, later, used for simulations in the “Results and discussion” section is built on two major pillars. The one is the glucose-insulin dynamics in dairy cows, which was modeled in [36] utilizing the Systems Biology Markup Language [37] and CellDesigner [38]. The other is the bovine estrous cycle, modeled by a system of differential equations (BovCycle) that quantifies reproductive hormones and other relevant compartments, representing follicles and corpora lutea [25, 26].

**Fig. 1:**
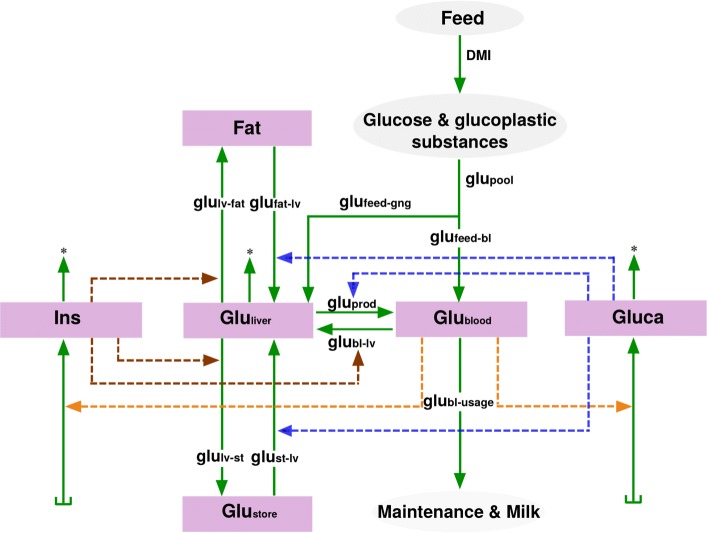
Schematic representation of the metabolic model. The pink boxes indicate the state variables of the model, gray ellipses indicate sources and sinks. The five compartments of the underlying ODE model are denoted by upper case letters; they have units of concentration or mass (see also Table 2). Rates are denoted by lower case letters; they have units of gram per day (see also Table 3)

**Fig. 2:**
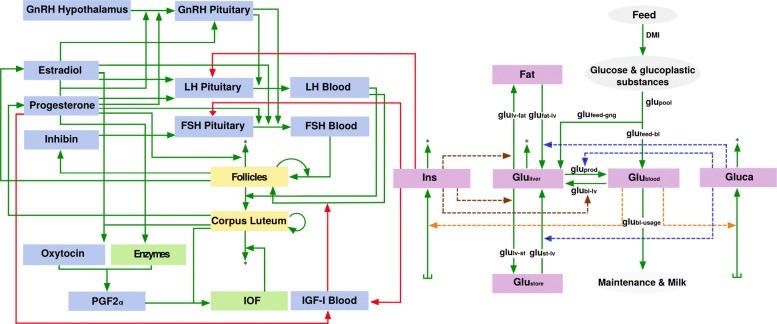
Schematic representation of the coupled metabolic-reproductive model. The coupled model links the metabolic model (right hand side) to the bovine estrous cycle model [26] (left hand side). Red arrows depict the sites where both models are coupled. Insulin acts on the site of anterior pituitary influencing LH and FSH release to the blood circulation. Insulin stimulates IGF-1 levels in the blood. Progesterone inhibits IGF-1 secretion which in turn decreases the responsiveness of follicular cells to LH

The model here consists entirely of ordinary differential equations (ODEs), which are solved for problem-specific initial conditions and parameter values. One half of the model (Fig. 1 and r.h.s. of Fig. 2) implements the mechanisms explained in [36], which allows for simulating the time-evolution of glucose and insulin for different dietary inputs in lactating as well as non-lactating cows. The other half (l.h.s. of Fig. 2) implements the biological feedback mechanisms between hypothalamus, pituitary gland and ovaries, which produces periodic estrous cycles of constant duration, similar to [26]. However, modifications needed to be implemented as the mechanisms suggested in [26] are not tailored to cows during pregnancy, calving and lactation. In these stages the interaction between hormones is somewhat different. To simulate the onset of lactation, oxytocin is included in the model; this hormone peaks during delivery [39], and it is required for milk ejection [40–42].

### Metabolic model

The metabolic model to be developed in this section is based on an improved version of the glucose-insulin model in [36]. It involves six components (*Glu*_*blood*_,*Glu*_*liver*_,*Glu*_*store*_,*Fat*,*Ins*,*Gluca*; see Table 2) and, as formulated here in terms of ODEs, their explicit interaction over time. Initial conditions are chosen based on the following calculation. For a cow of weight 600 kg and body condition score 3.5, the total body fat can be estimated by 25% of the total body weight [7, 43]. That is, 150 kg is taken as initial value for *Fat*. Typical physiological ranges for *Glu*_*blood*_, *Ins* and *Gluca* are listed in Table 1. As long as the initial values are within these ranges, they do not affect the performance of the model.

**Table 1:** Physiological ranges of blood plasma glucose, insulin and glucagon levels

Species	Range	Reference
*Glu*_*blood*_	0.39–0.59 g/L (2.22–3.30 mmol/L)	[99]
*Ins*	2–50 mU/L	[81, 100]
*Gluca*	50–120 ng/L	[81, 100]

**Table 2:** Species in the metabolic model

Name	Description	Initial value	Unit
*Glu*_*blood*_	Glucose concentration in the blood	0.48	g/L
*Glu*_*liver*_	Glucose generated in the liver	110	g
*Glu*_*store*_	Glucose stored as glycogen	535	g
*Fat*	Body fat	150	kg
*Ins*	Insulin concentration in the blood	15.5	mU/L
*Gluca*	Glucagon concentration in the blood	105	ng/L

The initial values are used to solve the differential equations

**Table 3:** Rates in the metabolic model

Name	Description	Unit
*glu*_*feed*−*bl*_	Glucose in the DMI available for direct absorption	g/d
*glu*_*feed*−*gng*_	Glucose generated from glucogenic substances in the DMI	g/d
*glu*_*bl*−*lv*_	Glucose absorbed from the blood into liver cells	g/d
*glu*_*st*−*lv*_	Glucose generated from glycogen (glycogenolysis)	g/d
*glu*_*lv*−*st*_	Glucose stored as glycogen (glycogenesis)	g/d
*glu*_*lv*−*fat*_	Glucose converted to triglycerides (lipogenesis)	g/d
*glu*_*fat*−*lv*_	Glucose synthesized from glycerol	g/d
*glu*_*prod*_	Glucose released from the liver to the blood	g/d
*glu*_*bl*−*usage*_	Glucose usage for maintenance and milk production	g/d
*glu*_*lv*−*usage*_	Glucose usage for liver metabolism	g/d
*ins*_*sec*_	Insulin secretion	mU/(L ·d)
*ins*_*deg*_	Insulin degradation	mU/(L ·d)
*gluca*_*sec*_	Glucagon secretion	ng/(L ·d)
*gluca*_*deg*_	Glucagon degradation	ng/(L ·d)

The model only involves the most basic mechanisms that regulate the flow of glucose through the body. It starts with the feed, continues with the digestive system and the blood, and ends up with glucose usage. Glucose and glucogenic substances are ingested with the dry matter intake (DMI). In the liver, the glucogenic substances are converted to glucose via gluconeogenesis. Glucose is used for maintanance and milk production, it is stored as glycogen or, after conversion, as fat. The compartments of the model and their interactions are illustrated in Fig. 1. Flows and regulatory mechanisms are summarized in Table 3 and explained in detail in the following subsections.

#### Feed intake

The first step involves the quantification of the amount of substances in the DMI that are either available for gluconeogenesis in the liver or directly absorbable as glucose into the blood. There exist empirical formulas that estimate the DMI needed to meet the energy requirements; these formulas are based on the cow’s body weight (BW) and the net energy (NE) of the diet; see, e.g., [7]. Throughout the paper, a standard cow with body weight 600 kg is considered, and the value for DMI of 11700 gram per day (g/d) is adopted from [36]. This value also results from a formula in [7], assuming a diet’s net energy of 1.32 Mcal/kg.^1^

Ruminants digestion involves fermentation, which makes consumption of a high-fiber diet possible and necessary [44, 45]. In the default setting, the fraction of glucose and glucogenic substances in the DMI, *glu*_*pool*_, is assumed to be 8% of the total DMI,

1 $$\begin{array}{@{}rcl@{}}  \mathit{glu_{pool}} = c_{0}\cdot \mathit{DMI}, \end{array} $$

where *c*_0_ is a mass-fraction parameter (with default value *c*_0_=0.08) that allows for varying the total amount of glucose and glucogenic substances that can be extracted from DMI. This fraction combines glucose precursor substances such as short chain fatty acids, which are converted to glucose in the liver by gluconeogenesis, as well as glucose that can directly be absorbed from the digestive tract into the blood [45–47]. In the cow, only very little glucose is available for direct absorption from the digestive tract [48]. From the total amount of glucose and glucogenic substances in the DMI (*glu*_*pool*_), the portion of glucose was estimated to be less than 10% [49–51], whereas up to 90% of *glu*_*pool*_ are glucogenic substances.

The flow of absorbable glucose that goes directly to the systemic circulation is incorporated into the model via the rate

2 $$\begin{array}{*{20}l} \mathit{glu_{feed-bl}} &= c_{1}\cdot\mathit{glu_{pool}}\,. \end{array} $$

The flow of glucose precursor substances that are converted to glucose by gluconeogenesis in the liver is incorporated into the model via the rate

3 $$\begin{array}{*{20}l} \mathit{glu_{feed-gng}} &= (1-c_{1})\cdot\mathit{glu_{pool}}\,. \end{array} $$

The default parameter value is *c*_1_=0.08 (cf. Table 4). It is assumed here that there is no loss from the glucose pool (the flows sum up to 1·*glu*_*pool*_), i.e., the processes take place with 100% efficiency. If some loss was included here, the simulation results presented further below would be the same but correspond to higher values of *c*_0_ (the amount of glucose and glucose precurser substances in the feed).

**Table 4:** Values of rate and effect parameters

Symbol	Value	Unit	Explanation
*c*_0_	0.08	–	Relative glucose content in the DMI
*c*_1_	0.08	–	Fraction of directly absorbable glucose
*c*_2_	84211	mU/(L ·d)	Rate constant for insulin secretion
*c*_3_	2105	1/d	Rate constant for insulin degradation
*c*_4_	70182	ng/(L ·d)	Rate constant for glucagon secretion
*c*_5_	350.87	1/d	Rate constant for glucagon degradation
*c*_6_	50	(g ·L)/(mU ·d)	Rate constant for glucose absorption from blood into liver cells
*c*_7_	180	L/(mU ·d)	Rate constant for glycogenesis
*c*_8_	0.22683	L/(mU ·d)	Rate constant for lipogenesis
*c*_9_	1350	(g ·L)/(ng ·d)	Rate constant for glycogenolysis
*c*_10_	3.5272	(g ·L)/(ng ·d)	Rate constant for gluconeogenesis
*c*_11_	0.0684	L/(ng ·d)	Rate constant for glucose release from the liver to the blood
*c*_12_	1000	g/d	Glucose usage for maintenance
*c*_13_	72	g/kg	Glucose usage for milk production
*c*_14_	5	1/d	Glucose usage for liver metabolism
*c*_17_	0.4	[IGF]/d	Basal IGF-1 synthesis rate in the blood
*c*_18_	1	[IGF]/d	P4- and insulin-regulated IGF-1 synthesis rate
*c*_19_	1.7	1/d	IGF-1 clearance rate
*c*_20_	3.49	1/d	Maximum effect of LH on follicular function
*c*_21_	1	[LH]	Maximum threshold of LH to stimulate follicular function
*c*_22_	3	–	Maximum effect of insulin on FSH synthesis in the pituitary
*c*_23_	1.05	–	Maximum effect of insulin on LH synthesis in the pituitary
*c*_24_	1.5	[Oxy]/d	Maximum rate of additional oxytocin synthesis during lactation
*c*_25_	0.0007	1/d^2^	Clearance of additional oxytocin during lactation
*V*	22.8	L	Extracellular volume of blood

#### Insulin and glucagon

The blood glucose concentration is maintained at normal levels primarily through the action of two hormones, namely insulin and glucagon. Any elevation in the blood glucose concentration leads to the production of insulin in the pancreatic beta cells. Insulin promotes glucose uptake in target cells, e.g., those in the liver, muscles and fat tissue, and it promotes the conversion of glucose to glycogen (glycogenesis) in the liver [52]. When the glucose blood concentration is low, the pancreatic alpha-cells produce glucagon. Glucagon increases the plasma glucose concentration by stimulating the generation of glucose from non-carbohydrate substrates (gluconeogenesis) and the breakdown of glycogen to glucose (glycogenolysis) in the liver [52]. In the model here, the dynamics of the blood insulin and glucagon concentrations are determined by their secretion rates (*ins*_*sec*_,*gluca*_*sec*_) and their degradation rates (*ins*_*deg*_,*gluca*_*deg*_),

4 $$\begin{array}{*{20}l}  \frac{d}{dt}\mathit{Ins} = ins_{sec} - ins_{deg},\quad \frac{d}{dt}\mathit{Gluca} = gluca_{sec} - gluca_{deg}, \end{array} $$

with linear degradation rates

5 $$\begin{array}{*{20}l}  \mathit{ins_{deg}} = c_{3}\cdot Ins,\quad \mathit{gluca_{deg}} = c_{5}\cdot Gluca. \end{array} $$

It is assumed that the insulin secretion rate increases when the glucose concentration in the blood is above a certain threshold value (*T*_1_ = 0.5 g/L = 2,77 mmol/L), whereas the glucagon secretion rate decreases whenever the glucose concentration in the blood is above that threshold value (*T*_2_ = 0.5 g/L = 2,77 mmol/L),

6 $$ \begin{aligned}  \mathit{ins_{sec}} &= c_{2}\cdot \mathit{H^{+} \left({Glu_{blood}}, T_{1}, 10\right)},\quad\\ \mathit{gluca_{sec}} &= c_{4}\cdot \mathit{H^{-} \left({Glu_{blood}}, T_{2}, 2\right)}. \end{aligned}  $$

The symbols *H*^+^ and *H*^−^ denote a positive and a negative Hill function,

$$ \begin{aligned} H^{+}(S,T,n):&=\frac{S^{n}}{S^{n}+T^{n}},\quad\\ H^{-}(S,T,n):&=\frac{T^{n}}{S^{n}+T^{n}}=1-H^{+}(S,T,n),  \end{aligned}  $$

which are used to model threshold-dependent stimulatory or inhibitory effects. Here, *S*≥0 denotes the substance, *T*≥0 the threshold, and *n*≥1 the Hill coefficient. A Hill function is a sigmoidal function between zero and one that switches at the threshold *S*=*T* from one level to the other with a slope specified by *n* and *T*. Threshold kinetics were selected to account for rapid adaptivity, which is an important mechanism to keep the plasma glucose concentration within the physiological range. There are no reference values for the individual rate constants *c*_2,3,4,5_, but their values were chosen such that a constant glucose blood concentration of *Glu*_*blood*_=*T*_1_=*T*_2_=0.5 g/L (resulting in a Hill function value of 0.5) would give rise to constant insulin and glucagon concentrations that are within the physiological range, namely 0.5·*c*_2_/*c*_3_=20 mU/L and 0.5·*c*_4_/*c*_5_=100 ng/L, respectively, compare Table 1.

#### Glucose production and storage in the liver

When the glucose blood level rises above a certain threshold (*T*_3_=0.45 g/L = 2,77 mmol/L), insulin promotes the absorption of glucose from the blood into liver cells (rate *glu*_*bl*−*lv*_),

7 $$\begin{array}{@{}rcl@{}}  \mathit{glu_{bl-lv} = c_{6}\cdot H^{+} \left({Glu_{blood}}, T_{3}, 10\right)\cdot Ins}\,. \end{array} $$

Insulin also stimulates the conversion of glucose available in the liver (*Glu*_*liver*_) to glycogen (glycogenesis rate *glu*_*lv*−*st*_). It is assumed here that this rate decreases when the cow produces more than a certain amount of milk (threshold *T*_4_=10 L) per day in order to make more glucose available for milk production. In addition, the rate *glu*_*lv*−*st*_ is switched off when the glycogen store, *Glu*_*store*_, reaches the maximal carrying capacity *K*=1000*g*^2^. The equation that describes this process is given by

8 $$ {\begin{aligned}  \mathit{glu_{lv-st} = c_{7}\cdot H^{-} \left({Milk}, T_{4}, 2\right)\cdot \left(1-\frac{Glu_{store}}{K}\right)\cdot Glu_{liver}\cdot Ins}\,. \end{aligned}}  $$

In addition, insulin promotes the absorption of glucose into fat cells and its conversion into triglycerides via lipogenesis. It is assumed here that this process is enhanced once the glycogen storage *Glu*_*store*_ is full (threshold *T*_6_=1000*g*). Again, similar to the glycogenesis rate *glu*_*lv*−*st*_, the rate is assumed to decrease when the cow produces more than a certain amount of milk (threshold *T*_5_=10 L) per day,

9 $$ {\begin{aligned} \mathit{glu_{lv-fat}= c_{8}\cdot H^{-} \left({Milk}, T_{5}, 1\right)\cdot H^{+} \left({Glu_{store}}, T_{6}, 10\right)\cdot Glu_{liver}\cdot Ins}\,. \end{aligned}}  $$

When nutritional supply with glucose is insufficient, the glucagon concentration increases and stimulates the breakdown of glycogen to glucose in the liver (glycogenolysis) to maintain blood glucose homeostasis [55]. This process is assumed to slow down when the glycogen store is below a certain threshold (*T*_7_=10*g*),

10 $$\begin{array}{@{}rcl@{}}  \mathit{glu_{st-lv} = c_{9}\cdot H^{+} \left(Glu_{store}, T_{7}, 10\right)\cdot {Gluca}}\,. \end{array} $$

In this case, i.e., when the glycogen store falls below a threshold (*T*_8_=10*g*), glucagon additionally stimulates the breakdown of lipids into glycerol and free fatty acids (lipolysis) in adipose tissue and the conversion of glycerol into glucose via gluconeogenesis in the liver. This rate is assumed to slowly decrease whenever the total body fat becomes smaller than a certain threshold (*T*_9_=150 kg),

11 $$ {\begin{aligned}  \mathit{glu_{fat-lv} = c_{10}\cdot H^{-} \left({Glu_{store}}, T_{8}, 10\right)\cdot H^{+} \left(Fat, T_{9}, 1\right)}\cdot Gluca\,. \end{aligned}}  $$

Finally, glucagon stimulates the release of glucose synthesized in the liver (*Glu*_*liver*_) into the blood,

12 $$\begin{array}{@{}rcl@{}}  \mathit{glu_{prod} = c_{11}\cdot \mathit{Glu_{liver}}\cdot Gluca}. \end{array} $$

In the equations above, threshold kinetics were selected for *Glu*_*store*_ to differentiate between full end empty store, without modifying the rates in dependence on the actual amount of glycogen in the store.

There are no reference values for the rate constants *c*_6_ to *c*_11_. They were fitted manually such that the simulation results qualitatively agree with the results reported in literature.

#### Glucose utilization

All organs and tissues of dairy cows use glucose, except adipose tissue which cannot directly convert glucose to fatty acids [45]. Glucose provides energy for maintenance and production. In the milk producing dairy cow, glucose utilization is dominated by the requirements of the mammary gland for milk synthesis [56]. These requirements increase rapidly right after parturition[57]. Glucose utilization is modeled here in terms of two different sink terms, one from *Glu*_*liver*_,

13 $$\begin{array}{@{}rcl@{}}  \mathit{glu_{lv-usage}} &= c_{14} \cdot \mathit{Glu_{liver}}, \end{array} $$

and one from *Glu*_*blood*_,

14 $$\begin{array}{@{}rcl@{}}  \mathit{glu_{bl-usage}} = c_{12}\cdot H^{+} \left({Glu_{blood}}, T_{10}, 10\right) + c_{13}\cdot \mathit{Milk}. \end{array} $$

The sink term from *Glu*_*blood*_ accounts for maintenance (1st term) and milk production (2nd term). Maintenance refers to glucose utilization by non-mammary tissues including brain and skeletal muscle, but excluding liver. For example, glucose that is stored in skeletal muscle as glycogen cannot be released back into the bloodstream due to the absence of glucose-6-phosphatase. It is assumed here that the glucose consumption for maintenance decreases when the glucose blood level drops below a certain threshold (*T*_10_=0.5 g/L = 2,77 mmol/L). The second term accounts for glucose utilized for milk production, including substance and energy. The variable *Milk* quantifies the daily milk yield in kg/day, whereas the parameter *c*_13_=72 g/kg [58] quantifies the amount of glucose (in gram) per kg of milk. Hence, the mammary glucose requirement in a cow with a daily milk yield of 40 kg would be about 3 kg per day. There is no reference value for the non-mammary glucose requirement, but according to the literature [56] this value should be significantly lower (here, *c*_12_=1 kg/day was chosen).

#### The system of differential equations

The final set of ordinary differential equations modeling the dynamics of the glucose exchange reads

15 $$\begin{array}{*{20}l}  V\cdot \frac{d}{dt}\mathit{Glu_{blood}} =\ & \mathit{glu_{feed-bl}} \,+\, \mathit{glu_{prod}} \,-\, \mathit{glu_{bl-lv}} \,-\, \mathit{glu_{bl-usage}}, \end{array} $$

16 $$\begin{array}{*{20}l} \frac{d}{dt}\mathit{Glu_{liver}} =\ & \mathit{glu_{feed-gng}} - \mathit{glu_{prod}} + \mathit{glu_{bl-lv}} - \mathit{glu_{lv-st}}\notag\\ & \,+\, \mathit{glu_{st-lv}} \,-\, \mathit{glu_{lv-fat}} \,+\, \mathit{glu_{fat-lv}}\,-\, \mathit{glu_{lv-usage}}, \end{array} $$

17 $$\begin{array}{*{20}l} \frac{d}{dt}\mathit{Glu_{store}} =\ & \mathit{glu_{lv-st}} - \mathit{glu_{st-lv}}, \end{array} $$

18 $$\begin{array}{*{20}l} \frac{d}{dt}\mathit{Fat} =\ & \mathit{glu_{lv-fat}} - \mathit{glu_{fat-lv}}, \\ \frac{d}{dt}\mathit{Ins} =\ & ins_{sec} - ins_{deg},\notag\\ \frac{d}{dt}\mathit{Gluca} =\ & gluca_{sec} - gluca_{deg},\notag \end{array} $$

where *V*=22.8 L is the extracellular volume of blood [36]. The ordinary differential equations were solved using the software MATLAB. The parameters and the initial values are listed in Tables 2, 4, and 5, respectively.

**Table 5:** Values of threshold parameters

Symbol	Value	Unit	Explanation
*T*_1_	0.5	g/L	Threshold of glucose in the blood to stimulate insulin secretion
*T*_2_	0.5	g/L	Threshold of glucose in the blood to inhibit glucagon secretion
*T*_3_	0.45	g/L	Threshold of glucose in the blood to stimulate the absorption of glucose into liver cells
*T*_4_	10	L	Threshold of milk to inhibit glycogenesis
*T*_5_	10	L	Threshold of milk to inhibit lipogenesis
*T*_6_	1000	g	Threshold of glygogen store to stimulate lipogenesis
*T*_7_	10	g	Threshold of glycogen store to stimulate glycogenolysis
*T*_8_	10	g	Threshold of glycogen store to stimulate gluconeogenesis
*T*_9_	150	kg	Threshold of fat to stimulate gluconeogenesis
*T*_10_	0.5	g/L	Threshold of glucose in the blood to stimulate non-mammary utilization
*T*_11_	0.3	[P4]	Threshold of P4 to inhibit IGF-1 synthesis
*T*_12_	15	mU/L	Threshold of insulin to stimulate IGF-1 synthesis
*T*_13_	0.171	[LH]	Threshold of LH to stimulate decrease of the follicular function
*T*_14_	0.5	[IGF]	Threshold of IGF-1 to stimulate the responsiveness of follicles to LH
*T*_15_	15	mU/L	Threshold of insulin to stimulate FSH synthesis
*T*_16_	16	mU/L	Threshold of insulin to stimulate LH synthesis

### A metabolic-reproductive model

Several studies have shown that the metabolic status has a large influence on growing cattle and on reproductive performance in dairy cows. During negative energy balance, which can be caused, e.g., by dietary restrictions or high milk yield, a remarkable change occurs in the levels of the metabolic components IGF-1, insulin, and glucose in the systemic circulation, which in turn influences the levels of reproductive hormones and follicular development [19–21]. The aim is to reproduce these observations by coupling the metabolic model and the reproductive model BovCycle introduced in [25, 26]. The initial values for the species in the BovCycle model are listed in Table 6. The flowchart for the coupled model is presented in Fig. 2. Detailed explanations of the coupling mechanisms are given in the three sections below.

**Table 6:** Initial values for species in the BovCycle model

No	Component	Initial value	Unit
1	GnRH in the hypothalamus	0.667	[GnRH]
2	GnRH in the pituitary	0.551	[GnRH]
3	FSH in the pituitary	0.316	[FSH]
4	FSH in the blood	0.395	[FSH]
5	LH in the pituitary	1	[LH]
6	LH in the blood	0.642	[LH]
7	Follicle	1	[Follicle]
8	PGF 2_*α*_	0.00506	[PGF 2_*α*_]
9	Corpus luteum	0	[CL]
10	Progesterone	0.004	[P4]
11	Estradiol	0.89	[E2]
12	Inhibin	0.826	[Inhibin]
13	Enzyme	0	[Enzyme]
14	Oxytocin (non-lactating case)	0.0183	[Oxy]
−	Oxytocin (lactating case)	2.5	[Oxy]
15	Insulin-like growth factor 1 (IGF-1)	0.48	[IGF]
16	Intra ovarian factor (IOF)	0.35	[IOF]

#### IGF-1 and insulin

**Fig. 3:**
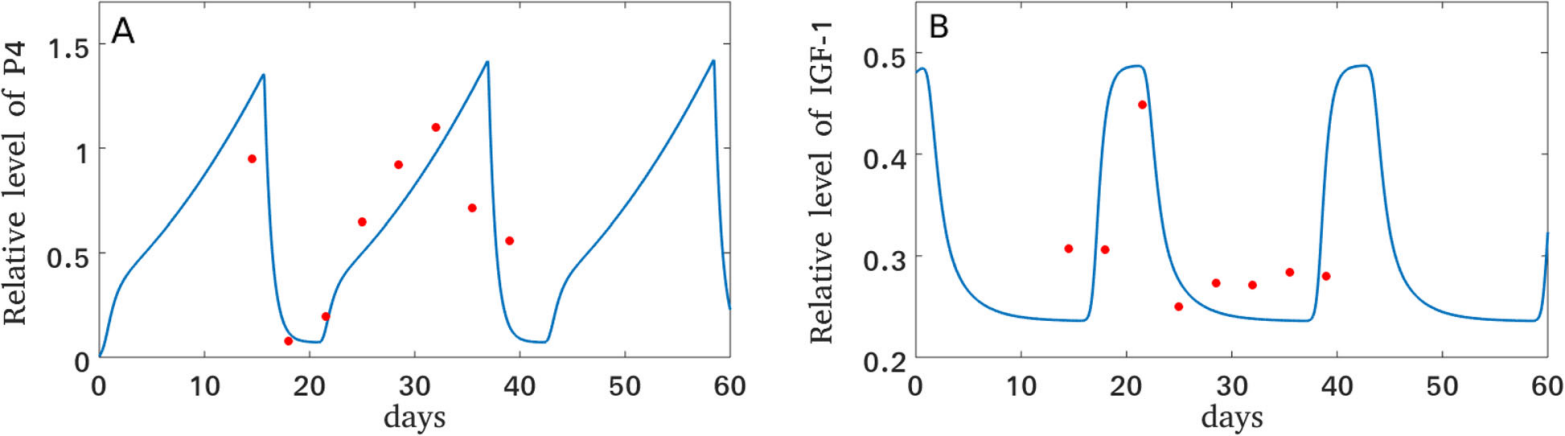
Changes in P4 and IGF-1 levels during the estrous cycle. Growth of P4 (**a**) is correlated to the decay of IGF-1 (**b**). Data of IGF-1 and P4 from 13 Holstein dairy cows (red dots) were collected and kindly provided by Kawashima et al. [59]

Kawashima et al. [59] reported that IGF-1 is positively correlated with the level of feed intake. The authors argue that the plasma IGF-1 concentration increases transiently during the follicular phase and decreases during the luteal phase of the estrous cycle, i.e., IGF-1 levels decrease when progesterone (P4) increases. On the other hand, IGF-1 is lowest during early lactation when there is no P4 in circulation, and highest in late lactation [60]. In particular, a decrease in blood insulin and glucose concentrations in postpartum cattle is associated with the decrease in IGF-I [21]. In addition, acute dietary restrictions reduce both insulin and IGF-1 concentrations in the blood [4, 61]. Even if these are only empirical observations and evidence for mechanistic relationships is missing, these observations are incorporated into the equation for IGF-1 as follows,

19 $$ {\begin{aligned} \frac{d}{dt}{IGF} = c_{17} + c_{18}\cdot H^{-} \left(P4, T_{11}, 4\right)\cdot H^{+} \left(Ins, T_{12}, 10\right) - c_{19}\cdot {IGF}, \end{aligned}}  $$

where *c*_17_ accounts for the basal IGF-1 synthesis rate. The rate constants *c*_17,18,19_ were determined such that the simulated IGF-1 concentrations match with the experimental data from 13 Holstein cows [59], see Fig. 3b. Moreover, in order to fit the simulated progesterone concentrations to the data (Fig. 3a), the basal P4 production rate had to be increased from *c*_*P*4_=0 in the original model [26] to *c*_*P*4_=0.1. This is consistent with reports about baseline progesterone levels [62].

A change in plasma IGF-1 has an impact on follicular cell development and responsiveness to hormonal signals. In particular, experimental studies demonstrated that reduced IGF-1 reduces ovarian responsiveness to LH stimulation [21, 63]. To include this mechanism in the model, the term in [26] that models the follicular cell responsiveness to LH,

$$ H^{+}({LH}_{Bld}) = c_{20} \cdot H^{+} \left(LH, T_{13}, 2\right),   $$

was improved as follows. The LH blood concentration that is required for an ovarian response (threshold *T*_13_) is made dependent on IGF-1,

20 $$\begin{array}{@{}rcl@{}}  T_{13}:= hm_{IGF} = c_{21}\cdot H^{-} \left(IGF, T_{14}, 2\right). \end{array} $$

Such a dependency was chosen because it allows for LH concentrations to increase in response to IGF-1 being below a certain threshold, *T*_14_. This mechanism is essential to ensure appropriate ovarian responses to IGF-1.

Insulin serves as a metabolic signal influencing the release of LH and FSH from the anterior pituitary into the blood [21, 64]. This mechanisms is included in the model by a stimulatory effect of insulin on the synthesis rates of LH and FSH. The equations for LH and FSH in [26] are changed to

21 $$\begin{array}{@{}rcl@{}} \frac{d}{dt}LH_{Pit} &= LH_{syn}\cdot hp^{LH}_{Ins} - LH_{rel}, \end{array} $$

22 $$\begin{array}{@{}rcl@{}} \frac{d}{dt}FSH_{Pit} &= FSH_{syn}\cdot hp^{FSH}_{Ins} - FSH_{rel}, \end{array} $$

where *LH*_*syn*_,*FSH*_*syn*_,*LH*_*rel*_, and *FSH*_*rel*_ are the synthesis and release rates of LH and FSH, respectively, as described in [26]. The Hill functions $hp^{LH}_{Ins}$ and $hp^{FSH}_{Ins}$ describe the influence of insulin on LH and FSH pituitary levels, respectively,

23 $$\begin{array}{@{}rcl@{}}  hp^{LH}_{Ins} &= c_{23}\cdot H^{+} \left(Ins, T_{16}, 10\right), \end{array} $$

24 $$\begin{array}{@{}rcl@{}} hp^{FSH}_{Ins} &= c_{22}\cdot H^{+} \left(Ins, T_{15}, 10\right). \end{array} $$

Hence, if insulin levels drop below a certain threshold (*T*_15_=*T*_16_=21 mU/L), the synthesis of LH and FSH halts.

#### Lactation

Pregnancy and calving are characterized by a complex interplay of hormones. One of these hormones is oxytocin. The release of this hormone and milk yield are positively correlated [41]. Overall as well as peak concentrations of oxytocin decrease over one ongoing lactation [65]; earlier studies reported similar dynamics [66–69]. According to measurements in those studies, peak concentrations of oxytocin during early lactation are more than twice the magnitude of those during late lactation.

The BovCycle model [26] does not capture changes in oxytocin concentrations during pregnancy and calving. To this end, the model was extended by introducing an additional term *Oxy*_*lac*_ into the equation of oxytocin,

25 $$\begin{array}{@{}rcl@{}}  \frac{d}{dt}Oxy = Oxy_{lac} + Oxy_{syn} - Oxy_{cle}, \end{array} $$

with

26 $$  Oxy_{lac} = c_{24}\cdot \exp(-c_{25}\cdot t^{2}).  $$

**Fig. 4:**
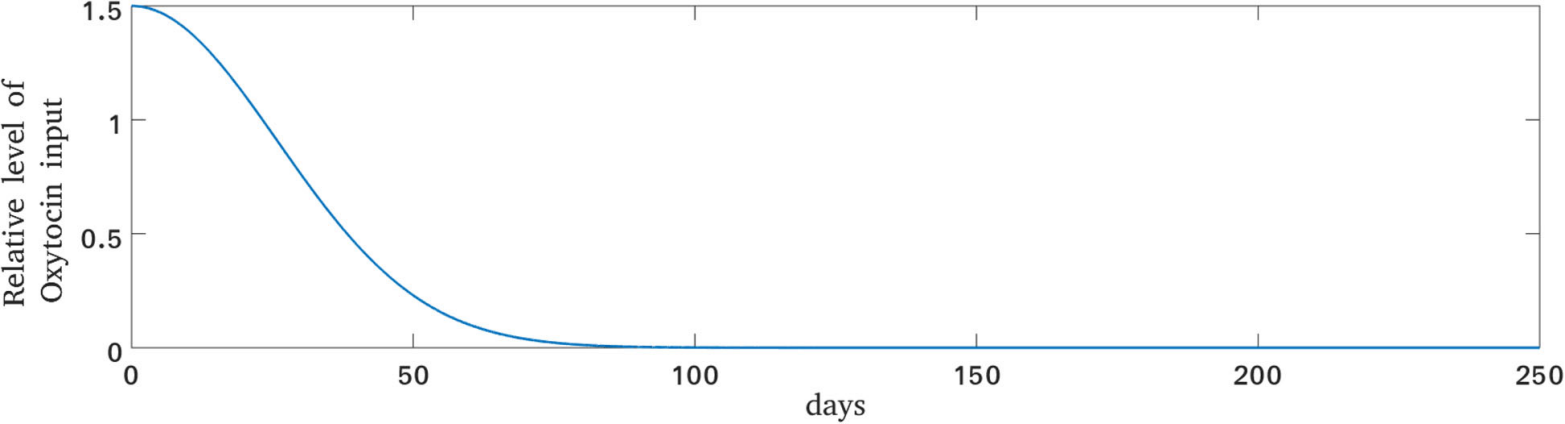
Modelled additional oxytocin during lactation. Plot of the additional time-dependent oxytocin source term during lactation as defined by Eq. (26)

This is the simplest form of a non-negative decreasing function, namely a Gaussian function, see Fig. 4. The parameter value *c*_25_=0.0007 determines the width of the curve and was adopted to the approximate length of the early lactation period, whereas the parameter value *c*_24_=1.5 was fitted so that *Oxy*(*t*) during early lactation is about twice as high as *Oxy*(*t*) during late lactation.

#### Reparametrization of the BovCycle model

The changes in the equations of the original BovCycle model [26] required changes of some of the original parameter values in order to be able to recover regular estrous cycles. In addition, the original BovCycle model [26] was challenged with the scenario of adding exogenous oxytocin early in the cycle. In a study by Donaldson et. al [70], it was shown that daily oxytocin injections to eight non-lactating cows starting on day two of the cycle reduced the estrous cycle length to nine days. The slow increase in plasma P4 concentration during the first five days of the cycle was not altered significantly, but plasma P4 concentrations decreased again to low values after day five. These results confirmed earlier studies [71, 72]. However, the original BovCycle model [26] did not reproduce these results. Hence, changes were made on parameters that describe the interaction of oxytocin and enzymes with prostaglandin *F*2_*α*_ and the interovarian factor such that the recalibrated model correctly reflects the effects of oxytocin administration on the length of the estrus cycle and plasma P4 concentrations. Parameters that required changes are listed in Table 7.

**Table 7:** Values of parameters that have been changed compared to [26]

Symbol	Value in [26]	New value	Unit	Explanation
*c*_*LH*_	12	2	1/d	LH clearance rate constant
*c*_*P*4_	0	0.1	[P4]/d	P4 baseline concentration in the blood
$\text {ex}^{CL}_{CL}$	2	30	–	Exponent of CL to stimulate self-growth
$\text {ex}^{Enz}_{PGF}$	5	1	–	Exponent of enzyme to stimulate prostaglandin F2 _*α*_ synthesis
$\text {ex}^{Oxy}_{PGF}$	2	10	–	Exponent of oxytocin to stimulate prostaglandin F2 _*α*_ synthesis
$\text {ex}^{P4}_{Enz}$	5	1	–	Exponent of P4 to stimulate enzyme synthesis
$\text {ex}^{PGF}_{IOF}$	5	10	–	Exponent of prostaglandin F2 _*α*_ to stimulate interovarian factor synthesis
$\text {ex}^{CL}_{IOF}$	10	1	–	Exponent of CL to stimulate interovarian factor synthesis
$T^{Follicle}_{FSH}$	0.57	1.497	[FSH]	Threshold of FSH to stimulate follicular function
$\mathrm {T}^{FSH}_{Follicle}$	0.22	0.322	[Follicle]	Threshold of follicular function to reduce FSH influence on follicular growth
$\mathrm {T}^{CL}_{CL}$	0.1	0.2807	[CL]	Threshold of CL to stimulate self-growth
$\mathrm {c}^{CL}_{CL}$	0.0334	0.0335	[CL]/d	Maximum rate of CL self-growth
$\mathrm {c}^{CL}_{LH}$	0.334	0.4	[CL]/d	Maximum rate of LH stimulated growth of CL

### Sensitivity analysis

Sensitivity analysis aims at determining the model input parameters which mostly contribute to a quantity of interest depending on the model output. Let us denote the model input parameter vector as $\mathbf {p}=(p_{1},\ldots,p_{d})\in {\mathbb R}^{d}$. The model here is an ordinary differential equation model of the form

$$ \mathbf{x}'(t)=f(\mathbf{x},\mathbf{p}),\quad \mathbf{x}(0)=\mathbf{x}_{0}\in{\mathbb R}^{n},   $$

and a quantity of interest, **y**, can be any observable depending on the model output **x**,

$$ \mathbf{y}=\mathbf{y}(\mathbf{x}(t,\mathbf{p})).   $$

This quantity can be for instance the value of a specific output variable *x*_*j*_ at a specific time point *t*, or the variance of *x*_*j*_ over a specific time interval. These are examples for scalar outputs. For the sake of simplicity, the study here is restricted to a scalar output *y*. The sensitivity of *y* with respect to input parameter *p*_*i*_ is given by

$$ S_{y}^{i}=\frac{\partial{y}}{\partial p_{i}}.   $$

To account for differences in physical units among variables and parameter, often relative sensitivities are used,

$$ {\hat S}_{y}^{i}=\frac{\partial{y}}{\partial p_{i}}\cdot\frac{|{p_{i}}|}{|{y}|}.   $$

If the exact derivative is difficult to compute, the sensitivity can be approximated by a finite difference scheme,

$$ S_{y}^{i}\approx\frac{y(\mathbf{x}(t,\mathbf{p}+\Delta e_{i}))-y(\mathbf{x}(t,\mathbf{p}))}{\Delta},   $$

where *Δ* is the size of the perturbation and *e*_*i*_ is a vector of the canonical base. Often, *Δ* is a relative perturbation, i.e., *Δ*=*ε*·*p*_*i*_ for some small number *ε* (e.g. *ε*=0.1) corresponds to a perturbation by 10%. In this case, the relative sensitivity is approximated by

27 $$ {\hat S}_{y}^{i}\approx\frac{y(\mathbf{x}(t,\mathbf{p}+\Delta e_{i}))-y(\mathbf{x}(t,\mathbf{p}))}{\epsilon\cdot |y(\mathbf{x}(t,\mathbf{p}))|}.  $$

This is a local sensitivity in the sense that it describes the influence of a specific local perturbation of parameter *p*_*i*_ on the model output. Sampling *Δ* or sampling pairs of input and output variables would allow for a global sensitivity analysis, but this is computationally much more demanding and the results are often difficult to interpret. For details on global sensitivity analysis, the reader is referred to [73].

For the metabolic-reproductive model presented here, sensitivity analysis is performed to determine the model parameters that are most important for the onset of luteal activity after calving. Hence, the observable *y* is chosen as the earliest time point at which the (relative) P4-level is larger than a threshold *T*_*P*4_=1,

$$ y(\mathbf{x}(t,\mathbf{p})):=\min_{t\geq 0}(P4(t)\geq T_{P4}).   $$

The results of this analysis are presented in the following section.

## Results and discussion

The aim of this study was to analyze the impact of supplied glucose, represented by the parameter *c*_0_, on the estrous cycle dynamics in both lactating and non-lactating cows. For this purpose, the model was simulated for different feeding scenarios, including short and long time dietary restrictions. For a cow of 600 kg BW, DMI at maintenance is set to its default value of 11.7 kg/d [36]. This is the reference value corresponding to 100% DMI throughout the following, and variations to this value are stated accordingly.

### Non-lactating cows

To model these cows, the value of *Milk* in Eq. (14) is set to zero. The numerical experiments for acute and chronic dietary restrictions are designed according to three experimental feeding studies from Mackey et al. [74], Murphy et al. [15] and Richards et al. [75]. Since these are studies in beef heifers and anestrus beef cows, respectively, the results are expected to agree only qualitatively, not necessarily quantitatively.

#### Varying the glucose content in the DMI

**Fig. 5:**
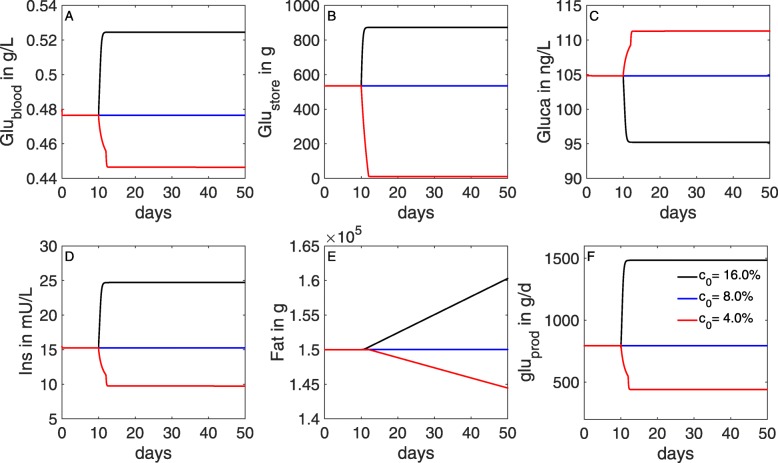
Simulated glucose and insulin dynamics in non-lactating cows for different values of glucose content in the DMI. The glucose content in the DMI is varied with *c*_0_={0.04,0.08,0.16}, corresponding to 4, 8, and 16%, whereby 8 *%* represents the amount required for maintenance. With higher/lower glucose content in the DMI, blood levels of glucose (**a**), insulin (**d**), stored glucose (**b**) and fat (**e**), and glucose production (**f**) increase/decrease over time. Glucagon (**c**) behaves inversely to the glucose blood level (**a**)

**Fig. 6:**
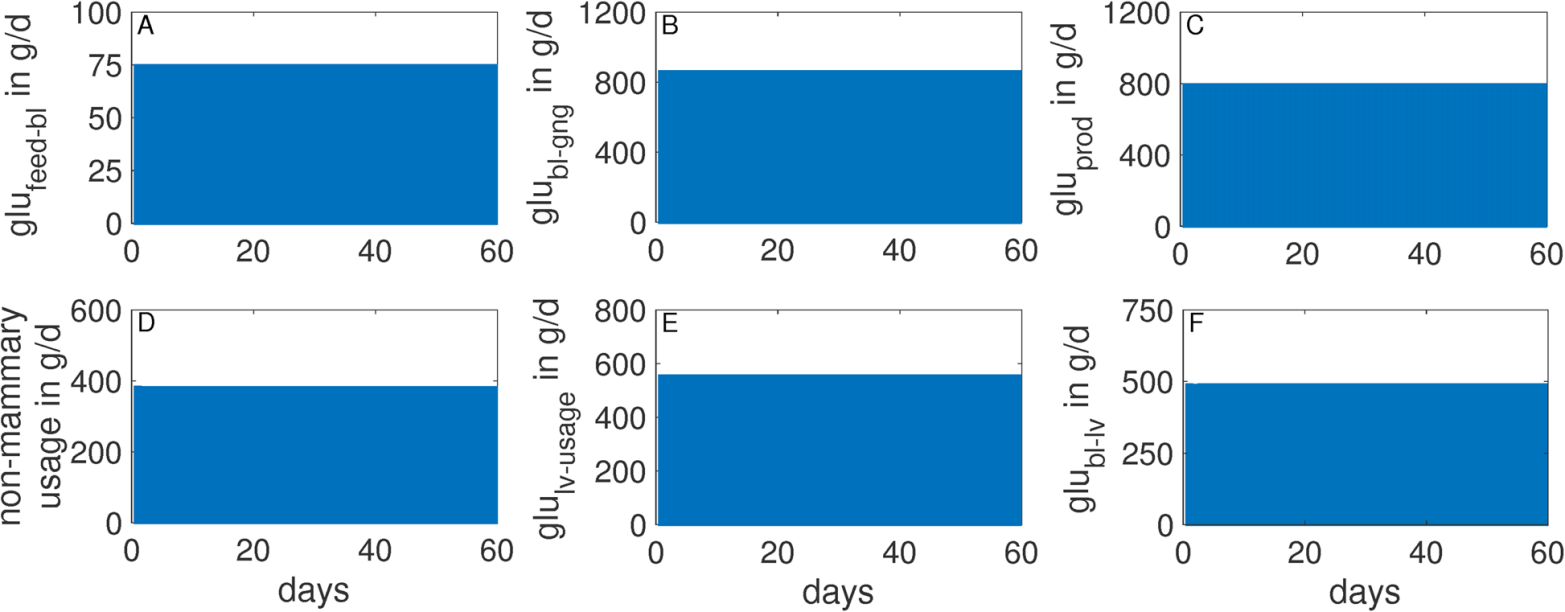
Simulated metabolic rates in non-lactating cows at maintenance. Glucose content in the DMI was fixed at 8%. The figure illustrates glucose input, storage, and usage in terms of the amount of glucose absorbed via the digestive tract (**a**), glucose generated from glucogenic substances in the feed (**b**), glucose released from the liver into the blood (**c**), glucose absorbed into liver cells (**f**), and glucose used for body maintenance (**d**) and for metabolic processes in the liver (**e**). At maintenance intake, the cow is able to cover the daily glucose requirement, which results in stable levels of glucose in the different compartments

The effect of varying glucose content in the DMI on the glucose-insulin dynamics is analyzed by changing the value of the parameter *c*_0_ (glucose content in the DMI) between 4%, 8% and 16%. Simulation results are presented in Figs. 5 and 6.

At maintenance intake, i.e. *c*_0_=0.08, the model calculates the non-mammary usage to be slightly less then 400 gram per day (Fig. 6d). This number is in qualitative agreement with Danfær *et al* [76], who estimated the amount of glucose required for maintenance in a non-lactating cow with a slightly lower body weight of 500 kg to be 290-380 gram per day. The amount of glucose absorbed from the digestive tract directly into the blood is calculated to be 75 g/d (Fig. 6a). The calculated amount of glucose released from the liver into the blood is about 800 g/d (Fig. 6c). This means that the total amount of glucose available in the blood is around 875 g/d, whereas the glucose uptake into liver cells (Fig. 6f) and the non-mammary usage (Fig. 6d) sum up to the same amount. This balance between input and consumption of glucose leads to stable glucose and insulin levels in the blood (Fig. 5a, d). In addition, this leads to stable glycogen and fat levels in the respective storage components (Fig. 5b, e).

With increasing glucose content in the DMI (*c*_0_=0.16), more glucogenic substances are available and lead to an increased gluconeogenesis [45]. This increases glucose and insulin concentrations in the blood, but they are still within their physiological range (Fig. 5a, d). Excess glucose in the system is stored as glycogen or fat reserves (Fig. 5b, e). When the glucose content in the DMI is decreased to 4%, blood glucose and insulin levels decrease towards their lower physiological bounds within two days (Fig. 5a, d), compare Table 1. As a result, the stored glycogen and the fat reserves (Fig. 5b, e) are reduced as well.

#### Acute nutritional restriction

To simulate the effect of acute nutritional restriction on the estrous cycle, a numerical experiment was designed according to the study of Mackey et al. [74], who reported about the effect of nutritional deprivation for a period of 13–15 days. Heifers with 406 ±5 kg body weight were allocated to a diet with a DMI of 1.2% of body weight for maintenance and then reduced to a diet with a DMI of 0.4% of body weight. In the model here, this reduction to 1/3 of the default diet corresponds to a reduction in the DMI from 11.7 kg/d to 3.84 kg/d.

**Fig. 7:**
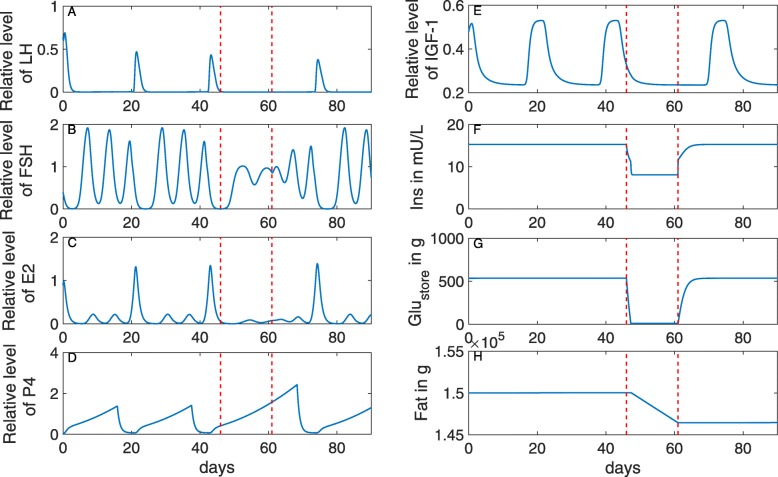
Effect of acute dietary restriction on the bovine estrous cycle in non-lactating dairy cows. On day 43, DMI is reduced from 100% (11.7 kg/d) to 33% (3.84 kg/d) for 15 days (the time period bounded by the two red lines). During the restriction period, one can observe a decrease of glucose in the store (**g**), insulin in the blood (**f**) and IGF-1 (**e**), an absence of LH pulses (**a**), and a decrease of amplitude in the FSH waves (**b**), leading to anovulation and failure in luteolysis with increasing P4 (**d**). The cycle re-starts soon after the end of the restriction period

This acute nutritional restriction is applied immediately after ovulation. The simulation results show increased levels of P4 (Fig. 7d), indicating a failure of luteolysis. Anovulation can be attributed to the absence of LH pulses (Fig. 7a) and lower FSH levels (Fig. 7b), as a result of decreased insulin levels (Fig. 7f). In addition, IGF-1 is decreased during the dietary restriction (Fig. 7e), which negatively influences the responsiveness of follicular cells to LH [20].

#### Chronic nutritional restriction

To simulate the effect of chronic nutritional restriction on the estrous cycle, numerical experiments were designed according to the studies of Murphy et al. [15] and Richards et al. [75]. Murphy et al. [15] examined the effect of chronic dietary restriction on the estrous cycle over 10 weeks. In this study, heifers with 375 ±5 kg body weight were allocated to a maintenance diet with an amount of DMI corresponding to 1.2% of the body weight and a reduced diet with 0.7% of the body weight. In the model here, this reduction to 58% of the maintenance diet corresponds to a reduction in the DMI from 11.7 kg/d to 6.79 kg/d. In the experiment by Richards et al. [75], multiparous non-lactating Hereford cows underwent a chronic nutritional restriction for 30 weeks. They were fed to lose 1% of their bodyweight weekly. After the restriction period, the diet was increased to 160% of the maintenance diet.

**Fig. 8:**
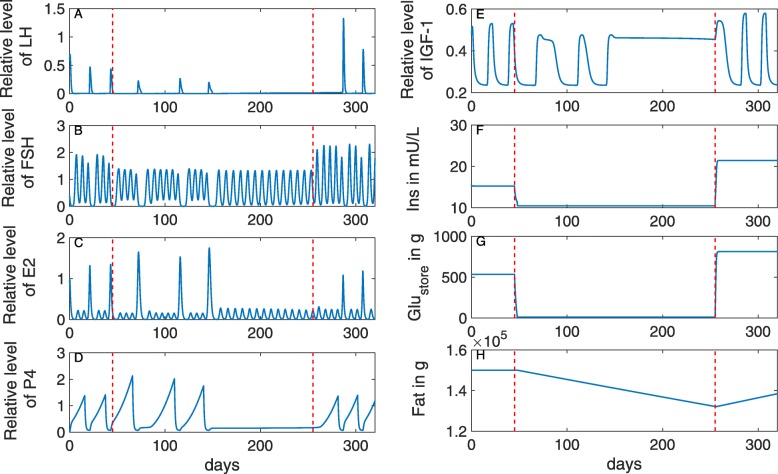
Effect of chronic dietary restriction on the bovine estrous cycles in non-lactating cows. DMI is reduced to 58% for 30 weeks (period between the red lines) and increased to 160% afterwards. During the restriction period, the glycogen store (**g**) and insulin in blood (**f**) decrease. LH (**a**), FSH (**b**) and IGF-1 (**e**) pulses decrease in frequency and amplitude, resulting in cessation of cyclicity after 15 weeks of feed restriction

The simulation was adapted to these two scenarios as follows. The nutritional restriction starts after ovulation. From then on, the model was simulated with 58% of the maintenance DMI within a time interval of 30 weeks. Simulation results (Fig. 8) show that the cow exhibits normal estrous cycles over a period of 15 weeks. During the chronic restriction period, the glycogen store (Fig. 8g) and the insulin in blood (Fig. 8f) decrease. LH (Fig. 8a), FSH (Fig. 8b) and IGF-1 (Fig. 8e) pulses decrease in frequency and amplitude, resulting in cessation of cyclicity after 15 weeks of feed restriction. The fat compartment loses around 10%. After 15 weeks, P4 decreases to a low level for the remaining 15 weeks, indicating the onset of anestrus. FSH and E2 exhibit changes in their wave patterns, that is, the number of waves per cycle increases. A similar tendency was observed in [15].

Murphy et al. [15] examined ultrasound data and serum P4 between week 6 and 9. They found no alteration in CL growth, whereas P4 in restricted cows was numerically higher than in cows on maintenance diet. No anestrus was observed in the first 10 weeks of the restriction period, which is in agreement with the simulation results.

During the first weeks of restriction in the experiment by Richards et al. [75], P4 concentration increased as well. After losing 24.0 ±0.9*%* of their initial body weight, cows had decreased luteal activity measured via P4, and cessation of the estrous cycle was observed in 54% of the cows after 26 weeks. The authors reported that estrous cycles were re-initiated by week 40 in 64% of the restricted cows, feeding 160% of maintenance diet. The model predicts re-initiation of cyclicity by week 32, feeding 160% of DMI at maintenance.

### Lactating cows

**Fig. 9:**
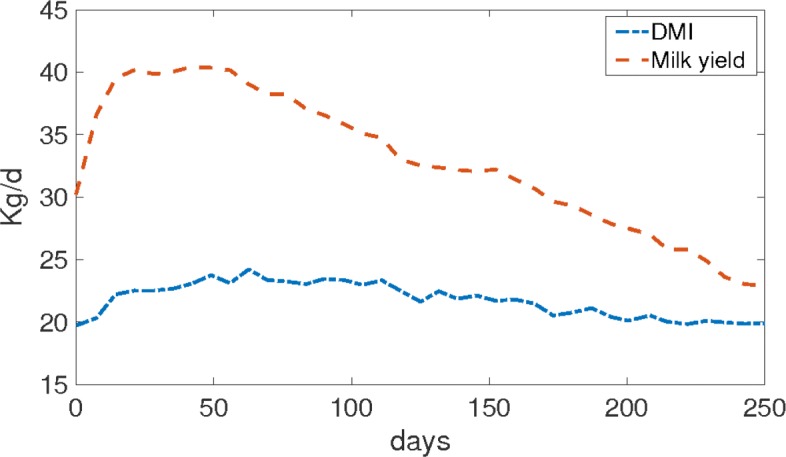
Model input data of DMI and milk. In this data, the highest milk yield (about 41 kg/d) can be observed 8 weeks postpartum. It coincides with the peak in the DMI (22 kg/d)

To investigate the effect of lactational metabolism and NEB on fertility hormones, different scenarios were simulated with the metabolic-reproductive model. As model input, interpolated time series data of DMI and milk yield from a study by Friggens et al. [77] were used, see Fig. 9. Each kilogram of milk produced requires around 72 gram glucose (parameter *c*_13_ in Eq. (14) [58]. Hence, the production of 41 kg milk per day requires about 3 kg of glucose per day. This was confirmed by Reynolds et al. [78], who predicted the glucose usage for milk to be between 2500 g/d and 3000 g/d. Milk production and the provided DMI in this study were 41 kg/d and 21 kg/d, respectively, averaged over 5 Holstein cows with an average body weight of 647 kg.

Energy balance is usually calculated as energy input minus output, requiring measurements of feed intake and energy output sources (milk, maintenance, activity, growth, and pregnancy)[79]. Alternatively, the energy balance can be calculated based on changes in the body reserves, using body weight and body condition score [79*,*^80^]. Since the model presented here does not explicitly calculate the body weight, the change in body fat is considered as an indicator of the energy balance,

28 $$\Delta_{Fat}= \mathit{glu_{lv-fat}} - \mathit{glu_{fat-lv}}.  $$

This approach was also used in [81].

#### Varying the glucose content in the DMI

**Fig. 10:**
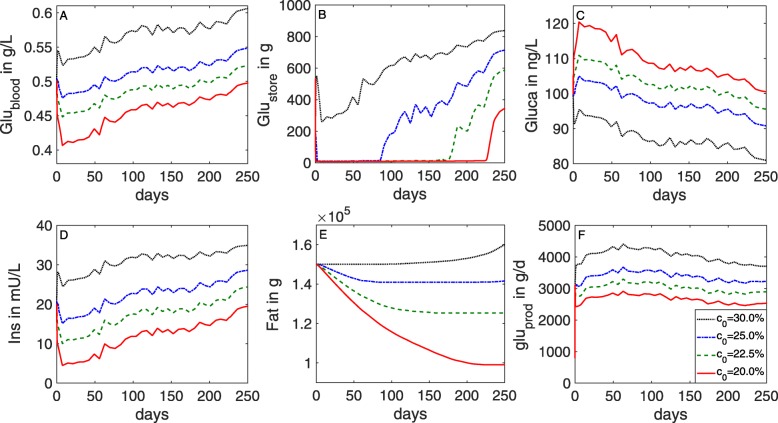
Simulated glucose and insulin levels in lactating dairy cows for different values of glucose content in the DMI. Time series data of milk yield and DMI from Holstein cows [77] are used as input for the model (**c**). Glucose and insulin dynamics were simulated with different glucose content in the DMI (*c*_0_={0.2,0.225,0.25,0.30}). When *c*_0_=0.2 (corresponding to 20% glucose content), glucose levels during peak milk drop towards the physiological limit (0.39 g/L) (**a**). In general, low amounts of glucose lead to a rapid depletion of the store (**b**), accompanied by a decrease in body fat (**e**), indicating a negative energy balance due to high milk production

**Fig. 11:**
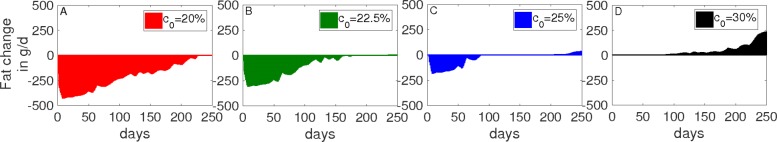
Simulated change in body fat as an indicator of energy balance in lactating dairy cows for different values of glucose content in the DMI. When *c*_0_=0.2, energy balance is negative throughout the lactation period (**a**). When *c*_0_=0.225 or higher, the period of negative energy balance becomes shorter (**b**,**c**). When *c*_0_=0.3, energy balance is positive throughout the lactation period (**d**)

**Fig. 12:**
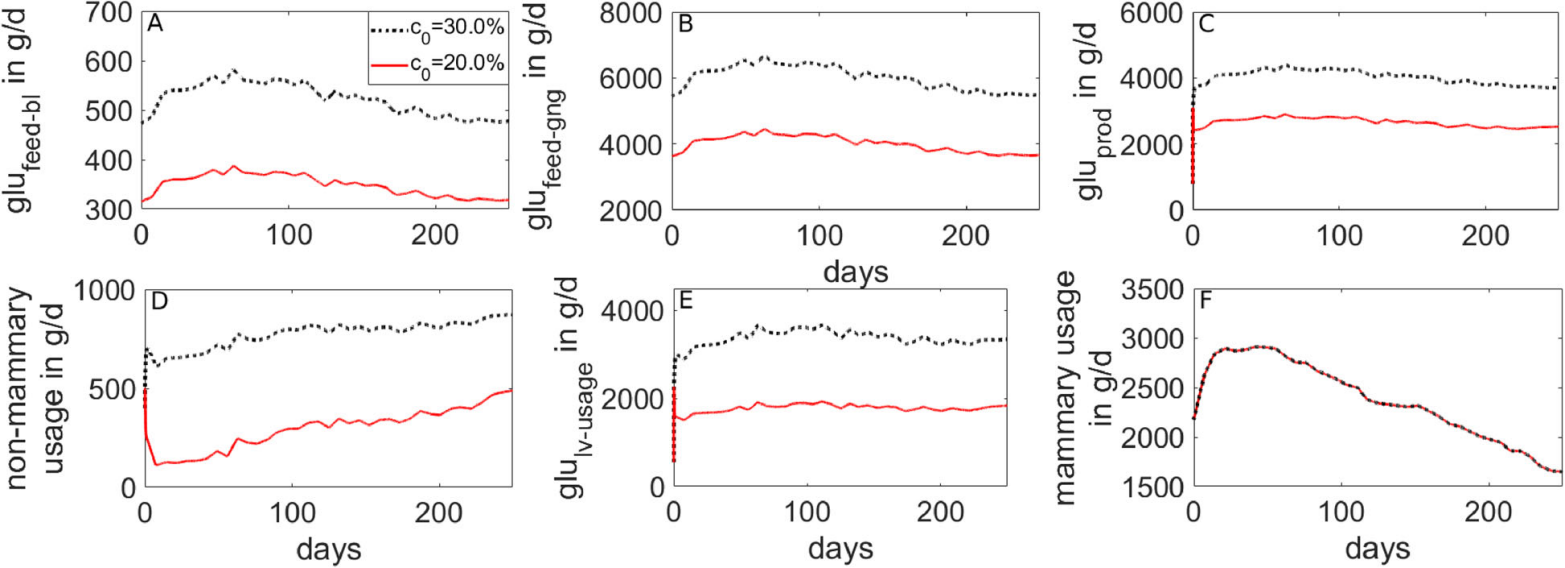
Simulated metabolic rates in a lactating cow for different values of glucose content in the DMI. Glucose content in the DMI was fixed at 20% (red line) or 30% (black line). During lactation, mammary glucose usage (**f**) gets prioritized compared to the non-mammary usage (**d**)

To explore the metabolic processes during lactation, simulations were performed for different values of glucose content in the DMI (parameter *c*_0_). The results are compared qualitatively with the studies by Elliot [82] and Reynolds et al. [78]. The changes in the glucose-insulin dynamics, body fat reserves, and metabolic rates are illustrated in Figs. 10, 11, and 12, respectively.

The simulation results clearly show a non-linear relationship between glucose content in the DMI and the values of glucose in blood and storage as well as insulin in blood at peak milk. Decreasing the glucose content in the DMI, starting from *c*_0_=0.3, first leads to a slow decrease in glucose and insulin levels, followed by a rapid decrease if *c*_0_ approaches the value 0.2.

For a high amount of glucose in the DMI (30%, *c*_0_=0.3), glucose and insulin levels in the blood are maintained within their physiological range (Fig. 10a, d). After the peak milk phase, the cow is even able to store glucose and fat (Fig. 10b, e). Consequently, the overall energy balance is positive throughout the lactation period (Fig. 11d). The model calculates the amount of glucose available in the circulation by direct absorption from the digestive tract (rate *glu*_*feed*−*bl*_) to be between 500 and 600 g/d (Fig. 12a). This is in agreement with Elliot [82], who estimated that for a cow with 600 kg BW and a milk yield of 40 kg/d, the amount of glucose absorbed from the digestive tract is around 600 g glucose per day.

For medium amounts of glucose in the DMI (22.5% or 25%), glucose and insulin levels are still kept within their physiological range (Fig. 10a, d), but the period of negative energy balance is prolonged (Fig. 11b, c).

If the amount of glucose in the DMI is decreased even further (20%, *c*_0_=0.2), one can observe an extended phase of negative energy balance with glucose and insulin dropping towards their lower physiological limits around peak milk (Fig. 10a, d). High demand and low input trigger the mobilization of body reserves, represented in the model by glycogen and fat in the store (Fig. 10b, e).

When *c*_0_ is varied between 0.2 and 0.3, the calculated amount of glucose released from the liver (*glu*_*prod*_) within the first 83 days post partum is 2500–4400 g/d (Fig. 12c). These numbers are in qualitative agreement with Reynolds et al. [78], who estimated the daily glucose production in the liver within the first 83 days post partum to be between 2700 and 3600 g/d. On can also observe that the mammary glucose usage gets prioritized compared to the non-mammary usage (Fig. 12f, d), and that this effect becomes more pronounced for low glucose diets.

#### The effect of changing glucose in the DMI on the estrous cycle

The glucose content in the DMI (parameter *c*_0_) has an effect on the estrous cycle. In the previous subsection, it was shown that decreasing *c*_0_ from 0.3 to 0.2 prolongs the phase of negative energy balance. A decrease in blood glucose and insulin concentrations is associated with a decrease in IGF-I [83*–*85]. As a consequence, elongated postpartum anestrus periods occur [86*–*89]. Similarly, Walsh et al. [5] resumed that NEB leads to low insulin concentrations in blood, which in turn prevents an increase in IGF-1 secretion, resulting in delayed resumption of ovarian cyclicity [90].

**Fig. 13:**
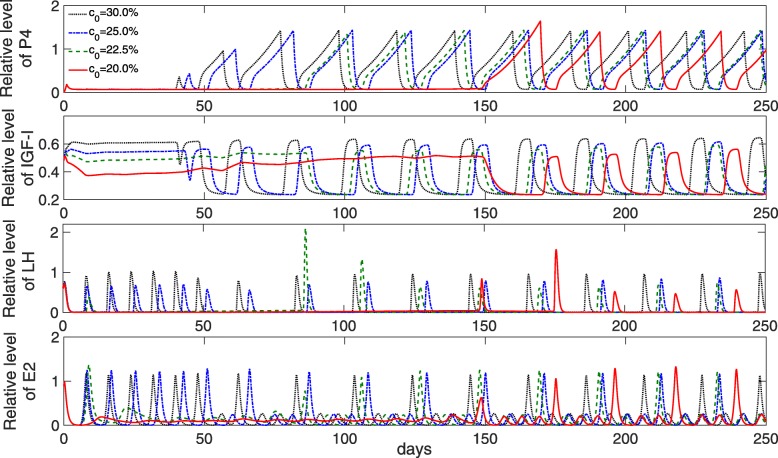
Simulated levels of P4, IGF-1, LH and estradiol during lactation for different values of glucose content in the DMI. Hormonal cycles were simulated over the lactation period for different fractions of glucose in DMI (parameter *c*_0_). A lower glucose content results in negative energy balance (Fig. 11), thereby prolonging the anestrus period. A higher glucose content results in an improved energy balance, which leads to increased insulin and IGF-1 levels and an earlier re-start of the estrous cycle

**Fig. 14:**
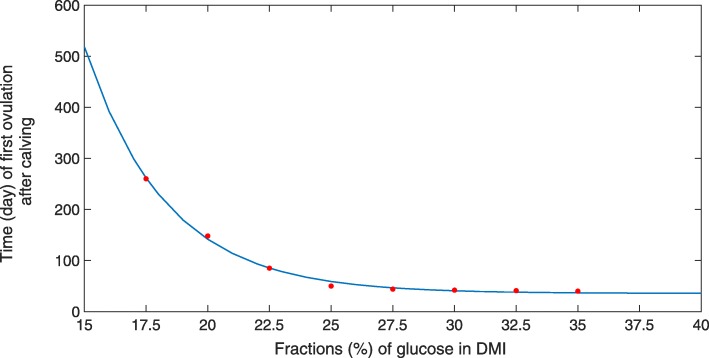
Effect of changing the glucose content in the DMI on the time of first ovulation after calving. Hormonal cycles were simulated over the lactation period for different fractions of glucose in DMI (parameter *c*_0_). Simulated data (red dots), which represents the estimated incidence of first ovulation, is determined by the time of first LH peak followed by an increase in progesterone production above baseline. The blue line represents the fitted curve *f*(*x*)=*a*· exp(−*b*·*x*)+*c* to the data with *a*=45581, *b*=0.30317, *c*=35.644. A lower glucose content results in a late ovulation, whereas a higher glucose content results in an early ovulation

**Fig. 15:**
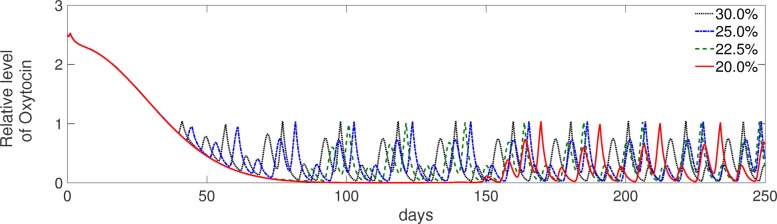
Simulated levels of oxytocin during lactation for different values of glucose content in the DMI. Levels of oxytocin, which are very high in early lactation, decrease with ongoing lactation and become cyclic again at the end of the anestrus period

The simulation results (Fig. 13) agree with those observations. Increasing the relative amount of glucose in the DMI from *c*_0_=0.2 to 0.3 increases the IGF-1 concentration. This stimulates the responsiveness of follicles to LH, thereby shortening the postpartum anestrus interval from about 150 to 40 days (Fig. 14). Accordingly, the oxytocin level becomes cyclic again at the end of the anestrus interval, after having significantly decreased over the postpartum period (Fig. 15).

The length of the postpartum anestrus in the simulations agrees with the literature. In studies based on postpartum progesterone profiles, it was demonstrated that 90 to 95% of post partum dairy cows have resumed ovarian cycles by day 50 after calving [91*–*93]. Hence, a postpartum dairy cow is considered ’normal’ if it has resumed ovarian cyclicity by day 50 post partum and continues cycling at regular intervals of approximately 21 days [94].

The simulations also show that estradiol levels at the beginning of the lactation period are within their normal range. This was confirmed by several studies. The authors in [75] found that restricted nutrition leads to anovulation but does not alter estradiol blood concentrations. Although ovulation and luteal development do not occur in anestrus cows, follicular growth is not totally impaired by restricted nutrient intake. In a review, Diskin et al. [10] suggested that NEB in early lactation does not affect the follicle population but does affect the ovulatory fate of the first dominant follicle. The authors summarized that low IGF-I and insulin cumulatively reduce follicular responsiveness to LH and ultimately suppress follicular oestradiol production.

There is evidence that a good management of the diet can reduce the incidence of abnormal estrous cycles [23*,*^24^*,*^27^]. Improving postpartum nutrition increases the blood concentration of insulin and IGF-I, which ultimately enhance LH pulsatility [19*,*^85^]. Higher IGF-1 levels during the first two weeks postpartum lead to an earlier re-start of the estrous cycle [5]. It was demonstrated in a study that providing a diet high in starch promotes an increased insulin release with a subsequent rise from 55% to 90% in the number of cows that ovulated within 50 days postpartum [24], a time interval that is considered to be an indicator for good reproductive performance [91]. In sum, resumption of cyclicity during lactation is crucial for good fertility in dairy farming. It can be influenced by feed intake, but also depends on many other factors such as uterine health, metabolic status, milk yield and overall condition.

#### The effect of changing model parameters on the estrous cycle

**Fig. 16:**
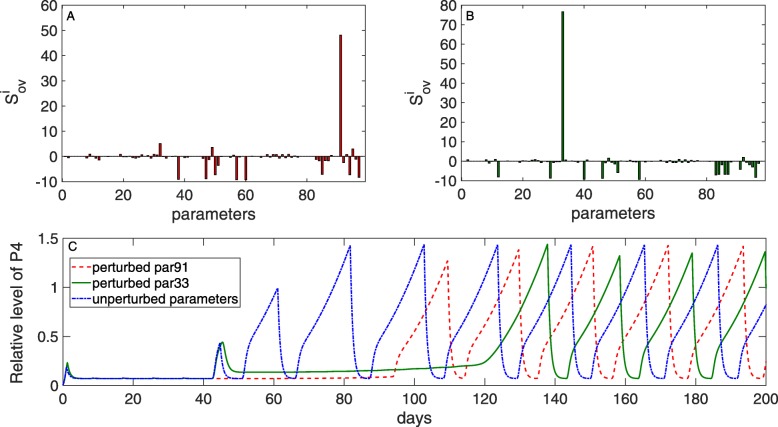
Sensitivity analysis results for the time of first ovulation post partum. A sensitivity analysis was performed to assess the influence of all model parameters on the time of first ovulation after calving as described by Eq. (27). **a** shows the change in the timepoint of first ovulation after perturbation of single parameters by +10%, whereas **b** shows the change in the timepoint of first ovulation after perturbation by -10%. Note that in the two subplots **a** and **b** only the numerator of ${\hat S}_{y}^{i}$ is plotted since the denominator is independent of the parameter index *i*. The two most influential parameters are *T*_1_ (parameter 91) and $T_{P4}^{Foll}$ (parameter 33). A change of the parameter *T*_1_ by +10% results in a later occurrence of ovulation (Fig. 16c). On the other hand, a decrease in the value of $T_{P4}^{Foll}$ by -10% stimulats the decay of follicular function, which causes a prolongation of the anovulatory period to day ≈120

A local sensitivity analysis as described in Eq. 27, was performed to assess the influence of all model parameters on the time of first ovulation after calving, characterized by the onset of luteal activity (increased P4 levels). Throughout the calculations, glucose content in the DMI was fixed at *c*_0_=0.25, which resulted in an onset of luteal activity at day 50 post partum. The parameters’ impact on the timepoint of ovulation is illustrated in Fig. 16. Figure 16a shows the change in the timepoint of first ovulation after perturbation of single parameters by +10%, whereas Fig. 16b shows the change in the timepoint of first ovulation after perturbation by -10%. Note that in the two subplots (A) and (B) only the numerator of ${\hat S}_{y}^{i}$ is plotted, since the denominator is independent of the parameter index *i*. The two most influential parameters are *T*_1_ (parameter number 91) and $T_{P4}^{Foll}$ (parameter 33, described in [26]). The first one describes the threshold of glucose in the blood to stimulate insulin secretion, while the second one is the threshold of P4 to stimulate decrease of follicular function. A change of the parameters 91 and 33 by +10% and -10%, respectively, results in a later occurrence of ovulation (Fig. 16c). Indeed, an increase in the value of *T*_1_ by 10% limits the secretion rate of insulin. As insulin influences the release of LH, LH pulses are suppressed, which delays the ovulation to day ≈90. On the other hand, a decrease in the value of $T_{P4}^{Foll}$ by -10% stimulates the decay of follicular function, which causes a prolongation of the anovulatory period to day ≈120.

## Conclusion

In the previous sections, the relationship between fertility and metabolism was explored based on two validated models [26*,*^36^]. These models were slightly modified and coupled to simulate the interplay of follicular development and its hormonal regulation with the glucose-insulin system. Information about the mechanistic interactions between fertility and metabolism, if taken straight from the literature, is sometimes contradictory and/or redundant. Therefore, only a small number of mechanisms was included, sufficient to realize the coupling of the two models.

With the coupled model, acute and chronic dietary restriction scenarios were simulated, intending to reproduce clinical study findings for non-lactating cows [74*,*^75^*,*^95^]. Furthermore, numerical experiments were run by varying the amount of DMI and the glucose content in the DMI for both lactating and non-lactating cows, and the effect of dietary restrictions on the estrous cycle was analyzed in lactating cows. The simulation results agree with the findings from the clinical studies, at least on a qualitative level.

The graphs presented here show the same qualitative behavior as the graphs supplied by McNamara and Shields [33]. This is not surprising since the model in [33] uses parts of the BovCycle model [25,26] and combines it with the more complicated Molly model. Despite using independent data sets (compared to the study by McNamara and Shields), the parameter values and trends came out very similarly.

The model here has also some limitations. Increasing (decreasing) the glucose content in the DMI, given by the parameter *c*_0_, results in the same simulation output as increasing (decreasing) total DMI, because only the product *c*_0_·*DMI* is contained in the model equations but not the individual factors. In reality, this is certainly not true. A way out would be to relate DMI directly or indirectly (e.g., via metabolic activity as in [36]) to one of the other variables. However, this would have complicated the model structure which, from the authors’ point of view, is not necessary for the modeling purpose in this paper.

Furthermore, the model presented here only describes processes in a single representative cow. In its current form, the model is not able to display inter- or intra-individual variability. However, since the implemented mechanisms are universal, variability could easily be included by adapting parameter values to individual measurements, once such measurements are available.

One could also criticize the model for its restriction to glucose as the only feed component. Hence, the protein content should be included in addition to glucose and fat to complete energetic composition of DMI. This would provide one with a more realistic nutrient supply, change of body composition and body weight as well as milk production and composition.

In addition, experimental research is gaining more and more insights into the effect of nutrition on follicular development. With an improved follicle model, similar to the one introduced in [96], further simulations can be conducted to explore the effect of nutrition on multiple follicles in more detail.

So far, it is fair to say that the model presented here is only a starting point. It will certainly be modified and improved in future. However, by conducting numerical simulations relying on it, it was confirmed that an appropriate nutritional intake is fundamental in mitigating the effects and the extension of NEB in order to reduce the incidence of metabolic disorders in high producing cows and to avoid subsequent fertility problems [1,5,8*,*^97^]. To understand the interaction between nutrition, metabolism and reproduction, a unified approach was followed, similar to [33*,*^98^], where these fields of interest are integrated in one mathematical framework. The model here, formulated in terms of differential equations, enables the user to explore the relationship between nutrition and reproduction by performing related parameter studies. The local sensitivity analysis with respect to the onset of luteal activity after calving is just one example for such an analysis, which can easily be extended to other quantities of interest.

## Reviewer’s comments

We thank the editor and the reviewers for their time and effort to handle our manuscript. We revised our manuscript according to their recommendations.

### Reviewer 2, Tin Pang, Heinrich-Heine University, Düsseldorf, Germany

#### Reviewer summary

The manuscript, submitted by Omari et al., proposes a new phenomenological model of a dairy cow that couples the existing glucose-insulin model and the estrous cycle model, which qualitatively describes the level of hormones and glucose in the cow under different conditions. This new model divides a cow into different "compartments", e.g., liver, blood, etc., with network-like interactions between the level of various hormones in blood and the level of glucose in different compartments. The authors also showed that the model predictions roughly match the experimental results, supporting the conceptual validity of the model. While previous modelling studies have quantified the interaction of hormones and glucose level in different compartments, the proposed model provides a better description at the network level, providing a stepping stone to better understanding of the trade-off between reproduction and milk production of dairy cows, and may serve the general interest of the research community.

#### Recommendations to authors

No major issues.

## Supplementary information

**Additional file 1** The Matlab code contains the three m-files BovSys_para.m, BovSys_equa.m and BovSys_run.m. To start simulations, one has to run BovSys_run.m, which guides the user through different simulation scenarios (lactating/non-lactating with different diets). In addition, there are four data files containing the data for IGF (Data_IGF_Hol.mat), P4 (Data_P4_Hol.mat), milk (ML.mat), and DMI (DM.mat), that are needed as input files to run the model.

## Data Availability

All data generated or analysed during this study are included in this published article and its supplementary information files.

## Footnotes

1 In [7], the following formula was proposed for growing, non-lactating Holstein heifers.

$$\begin{array}{@{}rcl@{}}  \mathit{DMI} = \big(-0.1128 + 0.2435\cdot\mathit{NE}_{M} - 0.0466\cdot\mathit{NE}_{M}^{2}\big)\cdot\frac{ \mathit{BW}^{0.75}}{\mathit{NE}_{M}}, \end{array} $$

where DMI is in (kg/d), BW is the body weight (kg) and *N**E*_*M*_ is net energy of diet for maintenance. NE recommendations are stated in the range between 1.24 and 1.55 Mcal/kg.

2 Berg et al. [53] estimated that 2% of the weight of the muscle tissue is formed by glycogen, and 10% of the liver weight. Is was also estimated that for a cow with 600 kg body-weight the mass of muscle, liver and kidney is around 280 kg, whereof 9 kg is liver weight [54]. According to these numbers, the liver stores about 900 g glycogen.

## References

[1] Chagas L, Bass J, Blache D, Burke C, Kay J, Lindsay D, Lucy M, Martin G, Meier S, Rhodes F (2007). Invited review: New perspectives on the roles of nutrition and metabolic priorities in the subfertility of high-producing dairy cows. J Dairy Sci.

[2] Wathes D, Pollott G, Johnson K, Richardson H, Cooke J (2014). Heifer fertility and carry over consequences for life time production in dairy and beef cattle. Animal.

[3] Cabrera V (2014). Economics of fertility in high-yielding dairy cows on confined tmr systems. Animal.

[4] Walsh S, Matthews D, Browne J, Forde N, Crowe M, Mihm M, Diskin M, Evans A (2012). Acute dietary restriction in heifers alters expression of genes regulating exposure and response to gonadotrophins and igf in dominant follicles. Animal Reprod Sci.

[5] Walsh S, Williams E, Evans A (2011). A review of the causes of poor fertility in high milk producing dairy cows. Animal Reprod Sci.

[6] Opsomer G (2015). Interaction between metabolic challenges and productivity in high yielding dairy cows. Japanese J Vet Res.

[7] Subcommittee on Dairy Cattle Nutrition, Committee on Animal Nutrition, National Research Council (2001). Nutrient Requirements of Dairy Cattle.

[8] Roche JF (2006). The effect of nutritional management of the dairy cow on reproductive efficiency. Animal Reprod Sci.

[9] Janovick N, Boisclair Y, Drackley J (2011). Prepartum dietary energy intake affects metabolism and health during the periparturient period in primiparous and multiparous holstein cows. J Dairy Sci.

[10] Diskin M, Mackey D, Roche J, Sreenan J (2003). Effects of nutrition and metabolic status on circulating hormones and ovarian follicle development in cattle. Animal Reprod Sci.

[11] Rawan A, Yoshioka S, Abe H, Acosta T (2015). Insulin-like growth factor-1 regulates the expression of luteinizing hormone receptor and steroid production in bovine granulosa cells. Reprod Domestic Animals.

[12] Short R, Bellows R (1971). Relationships among weight gains, age at puberty and reproductive performance in heifers. J Animal Sci.

[13] Day M, Imakawa K, Zalesky D, Kittok R, Kinder JE (1986). Effects of restriction of dietary energy intake during the prepubertal period on secretion of luteinizing hormone and responsiveness of the pituitary to luteinizing hormone-releasing hormone in heifers. J Animal Sci.

[14] Yelich J, Wettemann R, Dolezal H, Lusby K, Bishop D, Spicer L (1995). Effects of growth rate on carcass composition and lipid partitioning at puberty and growth hormone, insulin-like growth factor i, insulin, and metabolites before puberty in beef heifers. J Animal Sci.

[15] Murphy M, Enright W, Crowe M, McConnell K, Spicer L, Boland M, Roche J (1991). Effect of dietary intake on pattern of growth of dominant follicles during the oestrous cycle in beef heifers. J Reprod Fertil.

[16] Wiltbank J, Rowden W, Ingalls J, Zimmerman D (1964). Influence of post-partum energy level on reproductive performance of hereford cows restricted in energy intake prior to calving. J Animal Sci.

[17] Richards M, Spitzer J, Warner M (1986). Effect of varying levels of postpartum nutrition and body condition at calving on subsequent reproductive performance in beef cattle. J Animal Sci.

[18] Selk G, Wettemann R, Lusby K, Oltjen J, Mobley S, Rasby R, Garmendia J (1988). Relationships among weight change, body condition and reproductive performance of range beef cows. J Animal Sci.

[19] Beam S, Butler W (1999). Effects of energy balance on follicular development and first ovulation in postpartum dairy cows. J Reprod Fertil Suppl.

[20] Butler W (2000). Nutritional interactions with reproductive performance in dairy cattle. Animal Reproduction Science.

[21] Lucy M (2008). Functional differences in the growth hormone and insulin-like growth factor axis in cattle and pigs: implications for post-partum nutrition and reproduction. Reprod Domestic Animals.

[22] Butler WR (2003). Energy balance relationships with follicular development, ovulation and fertility in postpartum dairy cows. Livestock Prod Sci.

[23] Garnsworthy P, Gong J, Armstrong D, Newbold J, Marsden M, Richards S, Mann G, Sinclair K, Webb R (2008). Nutrition, metabolism, and fertility in dairy cows: 3. amino acids and ovarian function. J Dairy Sci.

[24] Gong J, Lee W, Garnsworthy P, Webb R (2002). Effect of dietary-induced increases in circulating insulin concentrations during the early postpartum period on reproductive function in dairy cows. Reproduction.

[25] Boer HMT, Stötzel C, Röblitz S, Deuflhard P, Veerkamp RF, Woelders H (2011). A simple mathematical model of the bovine estrous cycle: follicle development and endocrine interactions. J Theoret Biol.

[26] Stötzel C, Plöntzke J, Heuwieser W, Röblitz S (2012). Advances in modeling of the bovine estrous cycle: Synchronization with pgf2 *α*. Theriogenology.

[27] Pring SR, Owen M, King JR, Sinclair KD, Webb R, Flint AP, Garnsworthy PC (2012). A mathematical model of the bovine oestrous cycle: Simulating outcomes of dietary and pharmacological interventions. J Theoret Biol.

[28] Vetharaniam I, Peterson A, McNatty K, Soboleva T (2010). Modelling female reproductive function in farmed animals. Animal Reprod Sci.

[29] Meier S, Roche JR, Kolver ES, Boston RC (2009). A compartmental model describing changes in progesterone concentrations during the oestrous cycle. J Dairy Res.

[30] Martin O, Sauvant D (2007). Dynamic model of the lactating dairy cow metabolism. Animal.

[31] Baldwin RL (1995). Modeling Ruminant Digestion and Metabolism.

[32] Danfær A (1990). A dynamic model of nutrient digestion and metabolism in lactating dairy cows. Beretning fra Statens Husdyrbrugsforsøg.

[33] McNamara JP, Shields SL (2013). Reproduction during lactation of dairy cattle: integrating nutritional aspects of reproductive control in a systems research approach. Animal Frontiers.

[34] Martin O, Blanc F, Agabriel J, Disenhaus C, Dupont J, Ponsart C, Paccard P, Pires J, Freret S, Elis S (2012). A bovine reproductive physiology model to predict interactions between nutritional status and reproductive management. Proceedings of the 2012 Meeting of the Animal Science Modelling Group, Canadian J Animal Sci.

[35] Scaramuzzi R, Baird D, Campbell B, Driancourt M-A, Dupont J, Fortune J, Gilchrist R, Martin G, McNatty K, McNeilly A (2011). Regulation of folliculogenesis and the determination of ovulation rate in ruminants. Reprod, Fertil Develop.

[36] Berg M, Plöntzke J, Leonhard-Marek S, Elisabeth MK, Röblitz S (2017). A dynamic model to simulate potassium balance in dairy cows. J Dairy Sci.

[37] Hucka M, Finney A, Sauro H, Bolouri H, Doyle J, Kitano H (2003). The systems biology markup language (sbml): a medium for representation and exchange of biochemical network models. Bioinformatics.

[38] CellDesigner software. http://www.celldesigner.org. Accessed 10 July 2015.

[39] Landgraf R, Schulz J, Eulenberger K, Wilhelm J (1983). Plasma levels of oxytocin and vasopressin before, during and after parturition in cows. Experiment Clin Endocrinol Diabetes.

[40] Gorewit RC (1988). Lactation biology and methods of increasing efficiency. Designing Foods: Animal Product Options in the Marketplace.

[41] Negrão JA, Marnet P-G (2006). Milk yield, residual milk, oxytocin and cortisol release during machine milking in gir, gir × holstein and holstein cows. Reprod Nutrition Develop.

[42] Bruckmaier R, Blum J (1998). Oxytocin release and milk removal in ruminants. J Dairy Sci.

[43] Schröder U. J, Staufenbiel R (2006). Invited review: Methods to determine body fat reserves in the dairy cow with special regard to ultrasonographic measurement of backfat thickness. J Dairy Sci.

[44] Leng R (1970). Glucose synthesis in ruminants. Adv Vet Sci.

[45] Dijkstra J, Forbes JM, France J (2005). Quantitative Aspects of Ruminant Digestion and Metabolism.

[46] Kristensen NB, Danfær A, Agergaard N (1998). Absorption and metabolism of short-chain fatty acids in ruminants. Arch Animal Nutrition.

[47] Majdoub L, Vermorel M, Ortigues-Marty I (2003). Intraruminal propionate supplementation modifies hindlimb energy metabolism without changing the splanchnic release of glucose in growing lambs. Brit J Nutrition.

[48] Brockman RP (1983). Effects of insulin and glucose on the production and utilization of glucose in sheep (ovis aries). Comparative Biochem Physiol Part A: Physiol.

[49] Nafikov RA, Beitz DC (2007). Carbohydrate and lipid metabolism in farm animals. J Nutrition.

[50] Bergman E, Brockman R, Kaufman C (1974). Glucose metabolism in ruminants: comparison of whole-body turnover with production by gut, liver, and kidneys. Federation Proc.

[51] Young JW, Trott DR, Berger PJ, Schmidt SP, Smith JA (1974). Gluconeogenesis in ruminants: glucose kinetic parameters in calves under standardized conditions. J Nutrition.

[52] Brockman R (1978). Roles of glucagon and insulin in the regulation of metabolism in ruminants. a review. Canadian Veterinary J.

[53] Berg JM, Tymoczko JL, Stryer L (2002). Biochemistry.

[54] Pfuhl R, Bellmann O, Kuehn C, Teuscher F, Ender K, Wegner J (2007). Beef versus dairy cattle: a comparison of feed conversion, carcass composition, and meat quality. Arch Animal Breed.

[55] Habegger KM, Heppner KM, Geary N, Bartness TJ, Dimarchi R, Tschöp MH (2010). The metabolic actions of glucagon revisited. Nature Rev Endocrinol.

[56] Reynolds CK (2005). Glucose balance in cattle. Proceedings of the 16th Annual Florida Ruminant Nutrition Symposium.

[57] Aschenbach JR, Kristensen NB, Donkin SS, Hammon HM, Penner GB (2010). Gluconeogenesis in dairy cows: the secret of making sweet milk from sour dough. IUBMB Life.

[58] Kronfeld D (1982). Major metabolic determinants of milk volume, mammary efficiency, and spontaneous ketosis in dairy cows1. J Dairy Sci.

[59] Kawashima C, Kida K, Hayashi K-G, Montoya CA, Kaneko E, Matsunaga N, Shimizu T, Matsui M, Miyake Y-I, Schams D (2007). Changes in plasma metabolic hormone concentrations during the ovarian cycles of japanese black and holstein cattle. J Reprod Develop.

[60] Herbein JH, Aielle RJ, Eckler LI, Pearson RE, Akers RM (1985). Glucagon, insulin, growth hormone, and glucose concentrations in blood plasma of lactating dairy cows. J Dairy Sci.

[61] Thissen J-P, Underwood LE, Ketelslegers J-M (1999). Regulation of insulin-like growth factor–i in starvation and injury. Nutrition Rev.

[62] Bolaños J, Molina J, Forsberg M (1997). Effect of blood sampling and administration of acth on cortisol and progesterone levels in ovariectomized zebu cows (bos indicus). Acta Veterinaria Scandinavica.

[63] Stewart R, Spicer L, Hamilton T, Keefer B, Dawson L, Morgan G, Echternkamp S (1996). Levels of insulin-like growth factor (igf) binding proteins, luteinizing hormone and igf-i receptors, and steroids in dominant follicles during the first follicular wave in cattle exhibiting regular estrous cycles. Endocrinology.

[64] Monget P, Martin G (1997). Involvement of insulin-like growth factors in the interactions between nutrition and reproduction in female mammals. Human Reprod.

[65] Gorewit R, Wachs E, Sagi R, Merrill W (1983). Current concepts on the role of oxytocin in milk ejection. J Dairy Sci.

[66] Momongan V, Schmidt G (1970). Oxytocin levels in the plasma of holstein-friesian cows during milking with and without a premilking stimulus1. J Dairy Sci.

[67] Momongan VG (1969). The effect of milking stimulus and stage of lactation on the levels of oxytocin in the blood during the milking process.

[68] Randy H, Graf G (1973). Factors affecting endogenous plasma oxytocic activity in lactating holstein cows. J Dairy Sci.

[69] Randy H, Graf G, Bibb T. relationship between plasma oxytocin and reproductive status in lactating bovine. Amer Dairy Science Assoc. 1971; 54(5).

[70] Donaldson L, Bassett J, Thorburn G (1970). Peripheral plasma progesterone concentration of cows during puberty, oestrous cycles, pregnancy and lactation, and the effects of under-nutrition or exogenous oxytocin on progesterone concentration. J Endocrinol.

[71] Donaldson L, Takken A (1968). The effect of exogenous oxytocin on corpus luteum function in the cow. J Reprod Fertil.

[72] Donaldson L (1969). Effect of continued daily injections of oxytocin on oestrous cycle length and reproductive tract morphology in the cow. J Reprod Fertil.

[73] Iooss B, Lemaître P, Dellino G, Meloni C (2015). A Review on Global Sensitivity Analysis Methods. Uncertainty Management in Simulation- Optimization of Complex System, Vol. 59.

[74] Mackey DR, Sreenan JM, Roche JF, Diskin MG (1999). Effect of acute nutritional restriction on incidence of anovulation and periovulatory estradiol and gonadotropin concentrations in beef heifers. Biol Reprod.

[75] Richards M, Wettemann R, Schoenemann H (1989). Nutritional anestrus in beef cows: body weight change, body condition, luteinizing hormone in serum and ovarian activity. J Animal Sci.

[76] Danfær A (1994). Nutrient metabolism and utilization in the liver. Livestock Production Sci.

[77] Friggens N, Emmans G, Kyriazakis I, Oldham J, Lewis M (1998). Feed intake relative to stage of lactation for dairy cows consuming total mixed diets with a high or low ratio of concentrate to forage. J Dairy Sci.

[78] Reynolds C, Aikman P, Lupoli B, Humphries D, Beever D (2003). Splanchnic metabolism of dairy cows during the transition from late gestation through early lactation. J Dairy Sci.

[79] Thorup V, Edwards D, Friggens N (2012). On-farm estimation of energy balance in dairy cows using only frequent body weight measurements and body condition score. J Dairy Sci.

[80] Lovendahl P, Ridder C, Friggens NC (2010). Limits to prediction of energy balance from milk composition measures at individual cow level. J Dairy Sci.

[81] Weber C, Hametner C, Tuchscherer A, Losand B, Kanitz E, Otten W, Singh S, Bruckmaier R, Becker F, Kanitz W (2013). Variation in fat mobilization during early lactation differently affects feed intake, body condition, and lipid and glucose metabolism in high-yielding dairy cows. J Dairy Sci.

[82] Elliot J (1976). The glucose economy of the lactating dairy cow [metabolism]. Proceedings Cornell Nutrition Conference for Feed Manufacturers.

[83] Grummer RR (1995). Impact of changes in organic nutrient metabolism on feeding the transition dairy cow. J Animal Sci.

[84] Vicini JL, Buonomo FC, Veenhuizen JJ, Miller MA, Clemmons DR, Collier RJ (1991). Nutrient balance and stage of lactation affect responses of insulin, insulin-like growth factors i and ii, and insulin-like growth factor-binding protein 2 to somatotropin administration in dairy cows. J Nutrition.

[85] Lucy M (2000). Regulation of ovarian follicular growth by somatotropin and insulin-like growth factors in cattle1. J Dairy Sci.

[86] Butler W, Smith R (1989). Interrelationships between energy balance and postpartum reproductive function in dairy cattle. J Dairy Sci.

[87] Butler W, Everett R, Coppock C (1981). The relationships between energy balance, milk production and ovulation in postpartum holstein cows. J Animal Sci.

[88] Canfield R, Butler W (1990). Energy balance and pulsatile lh secretion in early postpartum dairy cattle. Domestic Animal Endocrinol.

[89] Staples C, Burke J, Thatcher W (1998). Influence of supplemental fats on reproductive tissues and performance of lactating cows1. J Dairy Sci.

[90] Gutierrez C, Gong J, Bramley T, Webb R (1999). Effects of genetic selection for milk yield on metabolic hormones and follicular development in postpartum dairy cattle. J Reprod Fertil Abst Ser.

[91] Opsomer G, Coryn M, Deluyker H, Kruif Ad (1998). An analysis of ovarian dysfunction in high yielding dairy cows after calving based on progesterone profiles. Rep Domestic Animals.

[92] Bulman DC, Lamming G (1978). Milk progesterone levels in relation to conception, repeat breeding and factors influencing acyclicity in dairy cows. J Reprod Fertil.

[93] Bulman DC, Wood P (1980). Abnormal patterns of ovarian activity in dairy cows and their relationships with reproductive performance. Animal Sci.

[94] Lamming G, Wathes DC, Peters A (1981). Endocrine patterns of the post-partum cow. J Reprod Fertil Suppl.

[95] Mackey D, Wylie A, Sreenan J, Roche J, Diskin M (2000). The effect of acute nutritional change on follicle wave turnover, gonadotropin, and steroid concentration in beef heifers. J Animal Sci.

[96] Lange A, Schwieger R, Plöntzke J, Schäfer S, Röblitz S (2019). Follicular competition in cows: the selection of dominant follicles as a synergistic effect. J Math Biol.

[97] Thatcher W, Santos J, Silvestre F, Kim I, Staples C (2010). Perspective on physiological/endocrine and nutritional factors influencing fertility in post-partum dairy cows. Reprod Domestic Animals.

[98] McNamara J (2012). Ruminant nutrition symposium: A systems approach to integrating genetics, nutrition, and metabolic efficiency in dairy cattle. J Animal Sci.

[99] Laboratory reference values. University of Leipzig, Department of Large Animal Medicine. http://www.vetmed.uni-leipzig.de/ik/wmedizin/labor/diagnostik/referenzw%erte/rind.htm. Accessed 10 July 2015.

[100] Weber C, Schäff C, Kautzsch U, Börner S, Erdmann S, Görs S, Röntgen M, Sauerwein H, Bruckmaier R, Metges C (2016). Insulin-dependent glucose metabolism in dairy cows with variable fat mobilization around calving. J Dairy Sci.

